# Hidden sequence specificity in loading of single-stranded RNAs onto *Drosophila* Argonautes

**DOI:** 10.1093/nar/gky1300

**Published:** 2018-12-27

**Authors:** Eling Goh, Katsutomo Okamura

**Affiliations:** 1 Temasek Life Sciences Laboratory, 1 Research Link, National University of Singapore 117604, Singapore; 2 School of Biological Sciences, Nanyang Technological University, 60 Nanyang Drive, Singapore 639798, Singapore

## Abstract

Argonaute proteins play important roles in gene regulation with small RNAs (sRNAs) serving as guides to targets. Argonautes are believed to bind sRNAs in a sequence non-specific manner. However, we recently discovered that Argonautes selectively load endogenous single-stranded (ss) RNAs, suggesting that Argonaute loading may conform to sequence specificity. To identify sequences preferred for Argonaute loading, we have developed HIgh-throughput Sequencing mediated Specificity Analysis (HISSA). HISSA allows massively parallel analysis of RNA binding efficiency by using randomized oligos in *in vitro* binding assays and quantifying RNAs by deep-sequencing. We chose *Drosophila* as a model system to take advantage of the presence of two biochemically distinct Argonautes, AGO1 and AGO2. Our results revealed AGO2 loading to be strongly favored by G-rich sequences. In contrast, AGO1 showed an enrichment of the ‘GAC’ motif in loaded species. Reanalysis of published sRNA sequencing data from fly tissues detected enrichment of the GAC motif in ssRNA-derived small RNAs in the immunopurified AGO1-complex under certain conditions, suggesting that the sequence preference of AGO1-loading may influence the repertoire of AGO1-bound endogenous sRNAs. Finally, we showed that human Ago2 also exhibited selectivity in loading ssRNAs in cell lysates. These findings may have implications for therapeutic ssRNA-mediated gene silencing.

## INTRODUCTION

Argonaute and Cas (Clustered Regularly Interspaced Short Palindromic Repeats-associated system) effectors provide flexible platforms whose target specificities could be artificially programmed by guide DNAs or RNAs. They both have potential as biotechnological tools that can artificially alter target gene expression or facilitate genome editing at specific sites (1,2). With little sequence specificity of these effector proteins for guide nucleic acids, these proteins can be programmed to specifically target a broad range of sequences. This feature of the effectors must be important for their ancestral roles to serve as natural defense systems against foreign agents including viruses and transposable elements (TEs) with various sequences that rapidly evolve (3–5). On the other hand, studies using unbiased large-scale methods revealed hidden sequence specificities of apparently ‘sequence non-specific’ RNA binding proteins (RBPs), raising the question of whether there exists any truly sequence non-specific RBP (6). Understanding the sequence specificity of Argonaute or Cas effectors may be important for developing effective biotechnological methods, such as RNA interference (RNAi) and genome-editing.

According to the dogma of microRNA (miRNA) biology, precursor RNA molecules that produce small RNAs loaded to the effector Argonaute have to be in the double-stranded (ds) configuration. Single-stranded (ss) RNA molecules are unstable in cells and their loading into Argonautes is assumed to be too inefficient to have significant regulatory activity (7). However, this dogma has been violated by the recent findings that cellular ssRNAs often populate effector complexes, leading to detectable regulatory activity by them (8).

A prominent class of ssRNA-derived small RNAs in the animal Argonaute complexes is hairpin loops of miRNA precursors (9,10). miRNA genes are generally transcribed by RNA polymerase II to produce primary transcripts (pri-miRNAs), which are subjected to a two-step processing mechanism involving two RNase III-class nucleases Drosha and Dicer (11). The products of Drosha are ∼60–70 nt hairpin-structured RNAs, known as precursor miRNAs (pre-miRNAs). Pre-miRNA hairpins are further cleaved by Dicer at the neck of the hairpin to release small RNA duplexes as well as single-stranded loop species. Duplexes are loaded into Argonautes after which one strand is discarded according to the strand selection rule forming mature miRNA-Ago complexes containing just one strand of the small RNA duplex (12,13). The remaining loop species were believed to be mere by-products with no functionality, but we and others showed that some of the loop species are loaded into Argonautes in *Drosophila* and mammals (9,10). Interestingly, loading of loops into Argonautes appears to occur in a selective manner, resulting in preferential loading of a small subset of miRNA loop species. Consistent with the notion that animal miRNA effector Argonautes prefer small RNAs carrying 5′ Uridine (U), most loaded loop species in the fly had 5′ U (9,14,15).

Here, we study the sequence specificity of ssRNA loading into Argonautes using typical miRNA and siRNA Argonautes, *Drosophila* AGO1 and AGO2 as model systems. We developed a high-throughput method to study relative loading efficiencies of ssRNAs with ∼1000s to 10 000s of different sequences in parallel. The results indicated that there exists hidden sequence specificity in ssRNA loading to the *Drosophila* Argonautes. In particular, fly AGO2 loading was facilitated by the presence of G-rich sequences, whereas sequences containing the GAC tri-nucleotides near the 5′ end were preferred for fly AGO1 loading. The results of high-throughput experiments could be verified by experiments using individually synthesized RNA oligos, indicating that our method faithfully reported the loading efficiency of individual ssRNA species. Effects of GAC on the natural AGO1-loaded population derived from ssRNA fragments could be detected under certain conditions, suggesting that the preferences revealed by HISSA may contribute to shaping the sequence composition of ssRNA-derived species in the AGO1 complex. Taken together, our results revealed that the classical 5′U is not the only nucleotide discrimination that could affect Argonaute loading, but suggested the presence of sequence motifs that are preferred for loading. Given the importance of ssRNA-mediated RNAi technology (16,17), our finding may have biotechnological implications, and provides a platform for further investigations on sequence specificity in Argonaute loading.

## MATERIALS AND METHODS

### *In vitro* loading assay

*In vitro* RNA loading assays were performed using cell lysate prepared from S2-R+ cells stably transfected with a FLAG-tagged AGO2 plasmid using a genomic fragment of the AGO2 locus (18,19). The experimental procedure was previously described (9). For each *in vitro* loading reaction, we used 160 μl of cell lysate and 2 × 10^6^ cpm of 5′ labeled RNA oligo. After incubation of the mixture for 1 h at 25°C, 700 μl PXL buffer (1× PBS (pH 7.4), 0.1% SDS, 0.5% sodium deoxycholate, 0.5% NP40) was added. FLAG-AGO2 and AGO1 protein complexes were immuno-purified from 400 μl of the loading reaction using 10 and 6 μg of mouse anti-FLAG (Wako, #012-22384) and rabbit anti-AGO1 (AbCam, #ab5070) bound on 60 and 36 μl of Dynabeads Protein G (Invitrogen), respectively. The same amounts of beads and normal IgG were used as negative controls. Precipitated RNA was extracted using Phenol/Chloroform/Isoamylalcohol and separated on 15% denaturing acrylamide gels, transferred to Hybond N+ nylon membranes (GE Healthcare). For *in vitro* loading assays with mammalian materials, we used HEK293T-A2 cells (20) stably transfected with a plasmid to express myc-tagged human Ago2. To obtain the plasmid, myc-tagged Ago2 cDNA sequence was amplified from pCDNA3-AGO2 (21) using AgeI_myc_F and MfeI_AGO2_R primers (Supplementary Table S4). The resulting fragment was inserted between Age I and Mfe I sites of modified pEM705 vector (20), whose multiple-cloning-site was modified by replacing the original 3′UTR with a fragment containing various restriction sites (Supplementary Table S4). HEK293-A2 lysate was prepared the same way as that of S2-R+. The loading conditions were also similar except that 4 × 10^6^ cpm of 5′ labeled RNA oligo was used per 160 μl cell lysate and incubation was carried at 37°C. Myc-AGO2 was pulled down using 10 μg of anti-myc bound on 60 μl of beads per 400 μl of the loading reaction.

### HISSA library construction

5′-5N HISSA libraries were constructed as previously described with some modifications (22). For 5′-8N HISSA libraries, we optimized the 3′ linker ligation condition and used a bridge DNA oligo that is complementary to 12nt of the 3′ end of mir-317 loop sequence and the 8nt of the 5′ end of the 3′-linker. We used a modified 5′ linker containing a 12nt UMI. Sequences of all oligos can be found in Supplementary Table S4. Sequencing was done on Hiseq400 at BGI (Hong Kong). For 5′-8N HISSA libraries, we optimized the 3′ linker ligation condition and used a bridge DNA oligo that is complementary to 12nt of the 3′ end of mir-317 loop sequence and the 8nt of the 5′ end of the 3′-linker. We used a modified 5′ linker containing a 12nt UMI. Sequences of all oligos can be found in Supplementary Table S4. Sequencing was done on Hiseq4000 at BGI (Hong Kong).

### Bioinformatics analysis of HISSA libraries

For all HISSA libraries, we first performed FastQC checks (https://www.bioinformatics.babraham.ac.uk/projects/fastqc/). Reads that did not contain the non-variable sequences for 5′-5N-mir-317-loop (TGAAATGCAAGCAAG) and 5′-8N-mir-317-loop (AATGCAAGCAAG) at the expected positions were excluded. For 5′-8N-HISSA, we further required the distance between ‘TGTAGC’ (RA5 linker before the two randomized nucleotides at the 3′ end) and ‘AATGCAAGCAAG’ (non-variable sequence of 5′-8N-mir-317-loop) to be 10nt. The 3′ linker sequence (Illumina default) was removed by fastX Toolkit (http://hannonlab.cshl.edu/fastx_toolkit). Reads with the same combination of the 12nt UMI and 5′-8N of the insert were collapsed and considered as one read assuming that they were results of PCR amplification. To eliminate reads derived from the same cDNA molecule but have different sequences due to PCR or sequencing error, we grouped reads with the same UMI in the 5′ linker region and within the groups, we looked for reads that are similar with one another (≤2nt mismatches). These reads were assumed to be a result of PCR amplification with errors and were further worked on. We identified the sequence with the highest read count as the sequence to represent the group and eliminated sequences of ≤4nt mismatches with respect to the representative sequence within the same UMI group. This was repeated until each UMI group was left with distinct (>4nt differences) insert sequences. To account for PCR and sequencing errors that occur in the UMI region, we grouped reads according to the 5′-8N region in the insert, and then applied the same error elimination procedure to the UMI sequence. In addition, the following were removed: (i) endogenous *mir-317 loop* sequences with two mismatches or less and (ii) crossed-contaminated sequences between 5′-5N and 5′-8N HISSA libraries.

The 5′-5N-HISSA libraries were analyzed using FASTAptamer (23) to calculate the enrichment factor of each sequence after AGO1- or FLAG-AGO2-IP. For the 5′-8N-HISSA libraries, the percentage of reads having each nucleotide (A, T, G, C) at each position (first to eighth) was calculated, and the ratio of the percentage between the AGO-IP and the corresponding input libraries were calculated and log2 transformed.

### Pairwise interaction analysis of 5′-8N HISSA data

In this analysis, all combinations of two nucleotides within the randomized regions were independently analyzed in 70 4-nt windows. For each 4-nt window, we first asked whether the combination of every two nucleotides showed statistically significant positive or negative effects on loading by linear regression analysis (*P* < 0.1). Those significant nucleotide pairs were then subjected to stepwise regression and the resultant linear coefficients were obtained. We averaged the coefficients of the same nucleotide combination in all 4-nt windows to compute the final coefficient of each interaction.

### Analysis of endogenous small RNAs in S2R+ AGO-IP libraries

AGO1-IP and FLAG-IP libraries previously constructed using S2R+ FLAG-HA-AGO2 cell line (9) were analyzed. The sequencing reads were collapsed and each sequence was expressed as a percentage of the total number of reads. The top ∼70% sequences of each IP library were extracted. Forty seven sequences were checked for the presence of ‘GTC’ in AGO1-IP and ∼550,000 sequences in AGO2-IP were used to generate sequence logo so as to observe the proportion of nucleotide ‘C’ for 21 positions.

### Computational pipeline for mRNA fragments analysis

The BED files of those previously mapped AGO1-IP and Input libraries (24) were used for downstream reads extraction and elimination. Reads lying within gene loci annotated in RefSeqGene (25) were extracted so as to eliminate all non-coding reads from sites such as telomeres and intergenic regions. Subsequently, reads belonging to all annotated miRNAs, siRNAs, piRNAs (for ovary libraries only) and repeat-masker were removed. siRNA annotations were as reported in previous studies (26–31). Loci of individual piRNAs residing in coding genes were downloaded from piRBase (32) and merged to obtain longer piRNA transcript loci. Additional siRNAs and piRNAs loci were obtained from Wen *et al.* (33). Repeat-masker annotation BED files were downloaded from UCSC Table browser (34,35). All multi-mappers, staggered-reads, and 21nt reads were also eliminated. At the end of all elimination steps, the remaining reads were treated as single-stranded mRNA fragments. For each library, 3nts-shifting frames were extracted up to the fifth position (first to third, second to fourth, third to fifth) of the mRNA fragments and a total of 64 (= 4^3^) 3nt-motifs with their respective read counts were obtained for each frame. The enrichment of the 3nt-motifs in AGO1 were obtained by normalizing the read counts in AGO1-IP to that of Input. *P*-values were calculated using Fisher's exact test in R.

## RESULTS

### Selective loading of phosphorylated ssRNA oligos onto Argonautes in fly cell extracts

**Figure 1.**
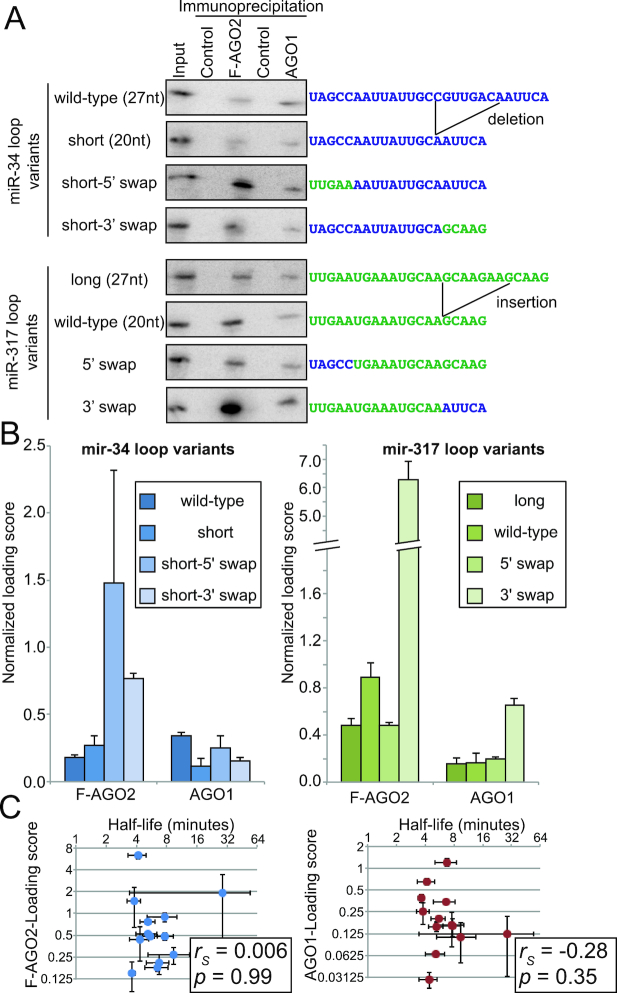
*In vitro* loading reveals distinct loading efficiencies of ssRNA species with different sequences. (**A**) Representative results of in vitro loading assays using mir-34 loop, mir-317 loop and their variants. 5′-^32^P-labeled synthetic RNA oligos with the indicated sequences were added to cell lysate prepared from S2-R+ cells stably transfected with FLAG-tagged AGO2 driven by the natural AGO2 promoter (19,26). The FLAG-AGO2 and endogenous AGO1 complexes were sequentially immuno-purified and co-precipitated RNA was analyzed on 15% denaturing acrylamide gels. Input is 0.01% of oligos introduced in an IP reaction. (**B**) Summary of the quantified results shown in (A). Loading scores were calculated by the following formula: (Signal intensity in the Argonaute-IP lane)*100/(Signal intensity in the Input lane). (**C**) Degradation rates of the 13 oligos used in this study were plotted against loading efficiency determined in (B). X- and Y-axes are shown in the log2 scale. No significant correlation was observed (Spearman correlation for F-AGO2: *rS* = −0.006 *P* = 0.99; for AGO1: *rS*= −0.28 *P* = 0.35). See Supplementary Figure S2 for the gel pictures and quantified data of degradation assays.

Our previous study demonstrated that preferential sorting of two miRNA loop species, miR-317-loop and miR-34-loop, to AGO2 and AGO1 could be recapitulated in an *in vitro* system (9). We attempted to quantify the loading preferences of RNA oligos harboring miR-317-loop and miR-34-loop sequences (Figure 1A; see ‘wild-type’ panels). The results of triplicates indicated that the *in vitro* system offers quantitatively reproducible results, which allowed us to perform confident statistical tests to distinguish the loading efficiencies of the two Argonaute complexes (Figure 1B, Average sorting ratios FLAG-AGO2/AGO1 for miR-34 loop and miR-317 loop were 0.52 and 6.55, respectively; Student's paired *t*-test *P* = 0.035). We verified the specificity of Argonaute purification by probing the membranes for endogenous small RNAs. An endo-siRNA (hp-CG4068B) and a miRNA (bantam) were enriched in the purified FLAG-AGO2 and AGO1 complexes, respectively, indicating the two complexes were specifically precipitated (Supplementary Figure S1).

Having confirmed the accuracy of the *in vitro* system, we sought to determine the range of loading efficiencies using a panel of RNA oligos with different sequences (Figure 1A and B). The best oligo was loaded at ∼2 or ∼4 times higher efficiency into the AGO1 or AGO2 complexes compared with the least efficient oligos, respectively (Figure 1B). Therefore, this confirms that Argonautes bind ssRNA species at distinct loading efficiencies depending on their sequences. We asked whether the length of oligos determines the preference as previously seen with duplex loading (15,18). The natural *mir-317* and *mir-34* hairpins produce loop species with the size of 20nt and 27nt as the major products (9). By introducing a 7nt deletion or insertion, we synthesized variants of these hairpin loops with different lengths (Figure 1A and B; short *mir-34* loop and long *mir-317* loop). While the deletion and the insertion affected loading rates, the length variants did not reverse the preferences. Therefore, the length of oligo did not explain the observed difference in the loading efficiency of the *mir-317* and *mir-34* loops.

To determine which part of ssRNA determines the loading rates, we generated variants based on *mir-317* and *mir-34* loop sequences by swapping the sequences of the first or last five nucleotides (Figure 1A and B; see ‘swap’ variants). These changes again influenced loading rates, however, none of these mutants supported a simple model whereby a single part of the RNA oligo could determine the loading rates. These results suggested that multiple parts throughout the small RNA have effects on loading efficiency.

### Degradation rates of RNA oligos are not a major determinant of loading efficiency

The generally lower loading efficiency of ssRNA species compared to duplex oligos is believed to stem from the faster degradation rates of ssRNAs in cells. Therefore, it was possible that the distinct loading efficiencies we observed with ssRNA oligos simply reflected their half-lives in the lysate, because more stable species are expected to have a higher probability to bind to Argonautes before they are degraded. To investigate the relationship between the loading efficiency and the degradation rate in the lysate, we measured half-lives of RNA oligos in the lysate (Figure 1C, Supplementary Figure S2). As expected, we observed quick degradation of all of the 12 RNA oligos with a median half-life of 5.53 min, suggesting that the vast majority of the introduced RNA molecules were degraded before they were loaded into Argonautes. However, when we plotted degradation rate against loading efficiency, we observed no significant correlation between the degradation rate and the efficiency of loading into F-AGO2 (Figure 1C, left panel, Spearman correlation *r_S_* = −0.006 *P* = 0.99) or AGO1 (Figure 1C, right panel, *r*_*S*_ = −0.28 *P* = 0.35).

These results indicated that degradation rates are not a major determinant of loading efficiency. Instead, other criteria must be determining the loading efficiency of each small RNA sequence.

### Sequence specificity in ssRNA loading into human Ago2

Since we found that *Drosophila* AGO1 exhibits sequence bias when loading ssRNAs, we were interested to test whether its major human counterpart Ago2 also shows sequence specificity when ssRNA is introduced to the lysate. To do so, we established a stable cell line that integrates a single copy of the plasmid sequence into the genomic DNA to express myc-tagged Ago2 (Supplementary Figure S3A) (20). We were able to successfully purify the Ago2 complex loaded with an unwound miRNA using anti-myc antibody after adding a synthetic miRNA duplex in the lysate, indicating that the lysate is capable of recapitulating small RNA duplex loading (Supplementary Figure S3B).

**Figure 2.**
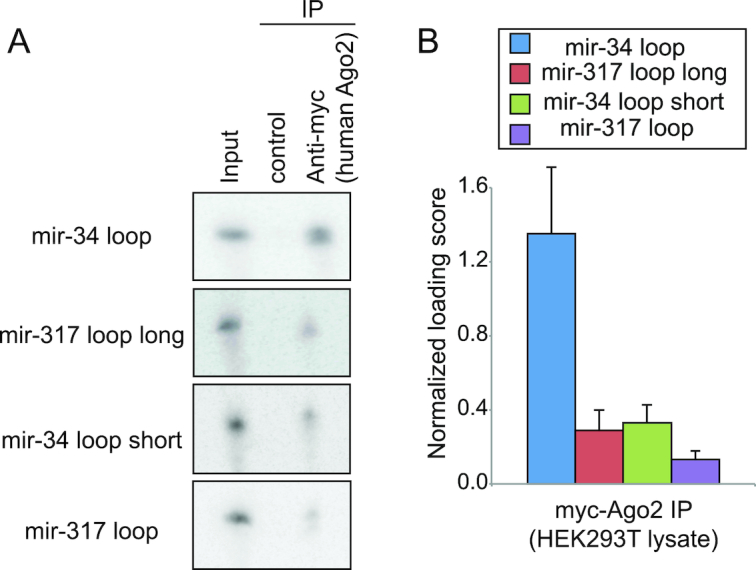
Selectivity in ssRNA loading onto human Ago2. (**A**) Representative results of *in vitro* loading assays using mir-34 loop, mir-317 loop and their variants. The sequences of the four oligos can be found in Figure 1A. Lysates were prepared from HEK293T cells expressing myc-hAgo2, and *in vitro* loading reactions were performed under a similar condition as Figure 1A. Input is 0.01% of oligos introduced in an IP reaction. (**B**) Summary of the quantified results shown in (A). The formula for Loading score calculation was same as Figure 1B.

Using this *in vitro* system, we performed *in vitro* loading assays using four ssRNA species (Figure 2A). Similar to *Drosophila* AGO1, human Ago2 could precipitate the *mir-34* loop oligo most efficiently among the four tested oligos (Figure 2B). These results suggested that non-random loading of ssRNA species into Argonautes might be a universal phenomenon that is broadly seen with many Argonaute proteins.

### Establishment of a massively parallel assay for ssRNA loading

**Figure 3.**
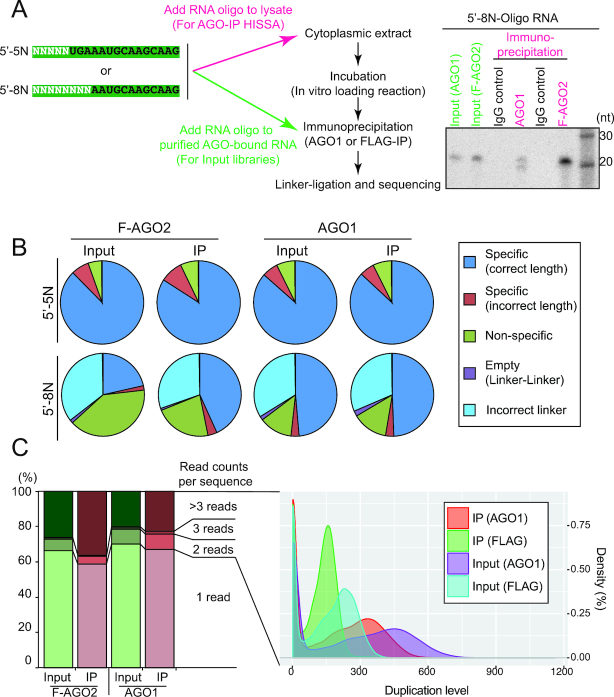
Establishment of a high-throughput *in vitro* loading assay. (**A**) Schematic representation of the experimental design. Partially randomized RNA oligos (first 5 or 8 nt for 5′-5N or 5′-8N, respectively) were radiolabeled at the 5′-phosphate, and introduced to the cytoplasmic extract. After the loading reaction, Argonaute complexes were isolated and extracted RNA was subjected to splinted-ligation and library construction. A typical gel picture of co-immunoprecipitated labeled RNA with randomized nucleotides were shown in the right panel. (**B**) Specificity of HISSA libraries. Reads in the sequenced libraries were grouped under the 5 categories as shown in the legend on the right side of the figure. Reads in the ‘Specific (correct length)’ category were used for further analyses. (**C**) Analysis of the read duplication level. UMI allowed us to detect the duplication events that occurred during the PCR amplification (see Materials and methods). The stacked columns show that ∼30–40% of reads exhibited evidence for amplification in each library. The right panel shows the distributions of duplication levels of individual sequences. Sequences with more than one read with identical UMI-insert combinations were used for the distribution plot. In some cases, hundreds of reads were derived from a single cDNA molecule.

The results above suggested that there are mechanisms selectively loading ssRNAs into Argonautes. To further study the mechanism, we sought to find features including sequences and potential secondary structures of ssRNAs that determine their loading efficiency. To this end, we set up an *in vitro* assay system that allows profiling of loading efficiency of individual ssRNA species in a massively parallel manner using complex mixtures of RNA oligos with partially randomized sequences (Figure 3A) (22). The fly system was chosen as a model, taking advantage of the fact that there are two biochemically distinct Argonautes showing clearly different ssRNA loading preferences (Figure 1A and B). We note that *Drosophila* AGO1 is more closely related to human AGO-clade Argonautes (Ago1-4) than to *Drosophila* AGO2 in the peptide sequence as well as the function as the major miRNA effector (7). Therefore, it is likely that *Drosophila* AGO1 and all the human Ago1-4 proteins share similar biochemical properties as seen with duplex loading mechanisms (36–39). We used *mir-317* loop variants whose sequences were changed to five or eight randomized nucleotides at the 5′ end (5′-5N or 5′-8N oligos, respectively; Figure 3A). Effects of 3′ sequence on loading is currently being studied, and the results will be discussed elsewhere. With the standard *in vitro* loading procedure, we were able to isolate species bound by AGO1 and FLAG-tagged AGO2 (F-AGO2) from the lysate. Partially randomized oligo nucleotides were specifically detected in Argonaute-IP samples but not in IgG controls, again demonstrating the specificity of the pulldown assays.

Because the immuno-purified Argonautes were expected to contain large amounts of endogenous small RNA species including miRNAs and siRNAs (5), we needed a method to enrich the introduced oligos when the deep sequencing libraries were constructed. We chose to use splinted-ligation to specifically add 3′ linker to the 3′ end of the introduced RNA oligo (40). By using this method, the ligation specificity and efficiency were high enough to construct small RNA libraries, and the majority of reads in the resulting libraries were derived from introduced oligos (Figure 3B and Supplementary Figure S4) (22). We applied the same experimental procedure to the ‘input’ RNA samples, which were prepared by mixing endogenous RNAs loaded to AGO1 or FLAG-AGO2 and randomized RNA oligos that were not subjected to the *in vitro* loading procedure (Figure 3A). Each HISSA library yielded ∼24–35 million reads by Illumina sequencing (Supplementary Table S1). Among those reads, reads with inserts having the correct constant region sequence flanked by the correct linker sequences constituted 21–88% of the libraries (Figure 3B).

The amounts of introduced RNA oligos in the immuno-precipitated samples were expected to be exceedingly low. Therefore, for 5′-8N HISSA, which produced more complex information with a large number of different sequences, we introduced Unique Molecular Identifiers (UMIs) to account for uneven PCR amplification of individual species (41). Using UMIs, we tried to eliminate amplification biases and sequencing errors (Figure 3C, see materials and methods). ∼30–40% of reads showed evidence for excessive amplification during PCR amplification, and the results indicated that hundreds of reads had been derived from a single cDNA molecule in some cases (Figure 3C). Although the fraction of amplified reads was small (∼30–40%), the high amplification levels (average: ∼40–73 times) of some reads were expected to affect read abundance quantification. Therefore, we used a newly developed computational pipeline to eliminate the effects of uneven PCR amplification (See Materials and Methods). Deduplicated read counts were used for the subsequent analyses. We used the input library as the baseline and calculated the enrichment factors in AGO-IP libraries in the following sections.

### HISSA reveals a preference of AGO2 loading for G-rich sequences

**Figure 4.**
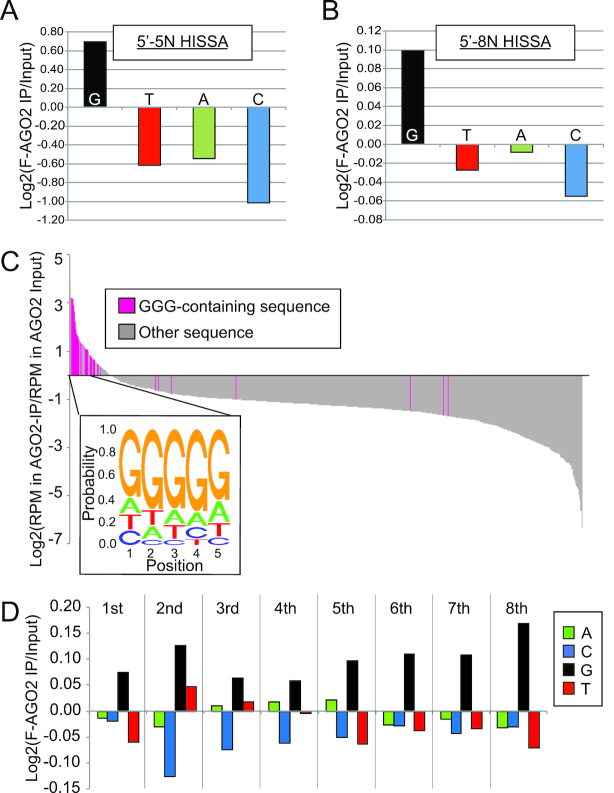
G-enrichment in AGO2-loaded population. Nucleotide enrichment of the randomized regions in the 5′-5N (**A**) and 5′-8N (**B**) FLAG-AGO2 HISSA libraries relative to the corresponding input libraries. The bar charts show the enrichment factors of the four nucleotides in each library set. (**C**) Summary of 5′-5N-HISSA results for AGO2. The enrichment factor after FLAG-AGO2-IP compared to the input library was calculated for each variant of the randomized 5 nucleotides, and sequences were sorted according to the enrichment factor. Bars corresponding to sequences that contained ‘GGG’ were highlighted with magenta. Magenta bars were enriched in the left side of the chart, indicating that sequences containing GGG were preferred for AGO2 loading. The sequence logo was generated using sequences that showed enrichment factor >1 and nucleotide probability at each position is shown. G was enriched at the first 5 positions. (**D**) Summary of 5′-8N-HISSA results for AGO2. The enrichment factor after FLAG-AGO2-IP was calculated for each nucleotide at each position as described in materials and methods. At all positions, we observed G to be the most preferred nucleotide for loading into FLAG-AGO2 throughout the randomized 8 positions of the 5′-8N oligo.

We first analyzed nucleotide compositions of the randomized region of the 5′-5N and 5′-8N oligos in the input and FLAG-AGO2-IP HISSA libraries (Figure 4A and B). When we calculated nucleotide compositions of randomized regions in the FLAG-AGO2-IP and corresponding input HISSA libraries, we observed a slight increase in the Guanine (G) content by FLAG-AGO2-IP, which may imply that G-rich ssRNAs are preferred for loading onto AGO2.

We decided to look for sequences preferred for AGO2-loading in this dataset. For the 5′-5N-HISSA data, with this level of complexity, it was possible to obtain quantitative information for the majority of the theoretical 1024 sequences (1021/1024 in Input and 1010/1024 in IP with >99 reads; 1024/1024 in both Input and IP with ≥1 read) (Supplementary Table S2). Therefore, we directly calculated enrichment factors of each sequence by dividing the relative read abundance in the FLAG-AGO2-HISSA library by that in the input library (Figure 4C). We looked for 3-nucleotide motifs enriched in FLAG-AGO2-HISSA by using the Bind-n-Seq platform (42,43). This analysis identified ‘GGG’ as the most enriched motif, explaining overall G-enrichment in the FLAG-AGO2-HISSA libraries (Supplementary Table S2). When the overall distribution of the FLAG-AGO2-IP enrichment factors for GGG-containing sequences was examined, >70% of GGG-containing sequences (28 out of 38) ranked within top ∼5% of all sequences (Figure 4C). The sequence logo drawn using the top ∼80 sequences showed clear enrichment of G at all positions, suggesting that G-rich sequences were preferred for loading onto AGO2.

For 5′-8N-HISSA, we were not able to compare individual sequences, because there were too few reads for each sequence due to the high complexity of the initial population, which was expected to contain >60,000 different sequences. Therefore, we analyzed how the four nucleotides at each position were enriched or depleted after AGO2-IP compared to the input population. We calculated enrichment factor of each nucleotide at each position of the five or eight nucleotides that were randomized in 5′-5N- and 5′-8N-HISSA library sets (Figure 4D, Supplementary Table S2). We found that G was enriched after AGO2-IP at all positions, again reaching the same conclusion that G is a preferred nucleotide for AGO2-loading in a position-independent manner.

Coincidentally, a G-rich sequence was also identified as a preferred sequence for AGO2-binding in a previous CLIP study (44). While that study did not distinguish between RNA species loaded as guide molecules and those bound as target RNAs, our results suggested that AGO2 may have an intrinsic property in preferring G-rich sequences (Supplementary Figure S8A).

### HISSA unveils a hidden motif that promotes AGO1 loading

**Figure 5.**
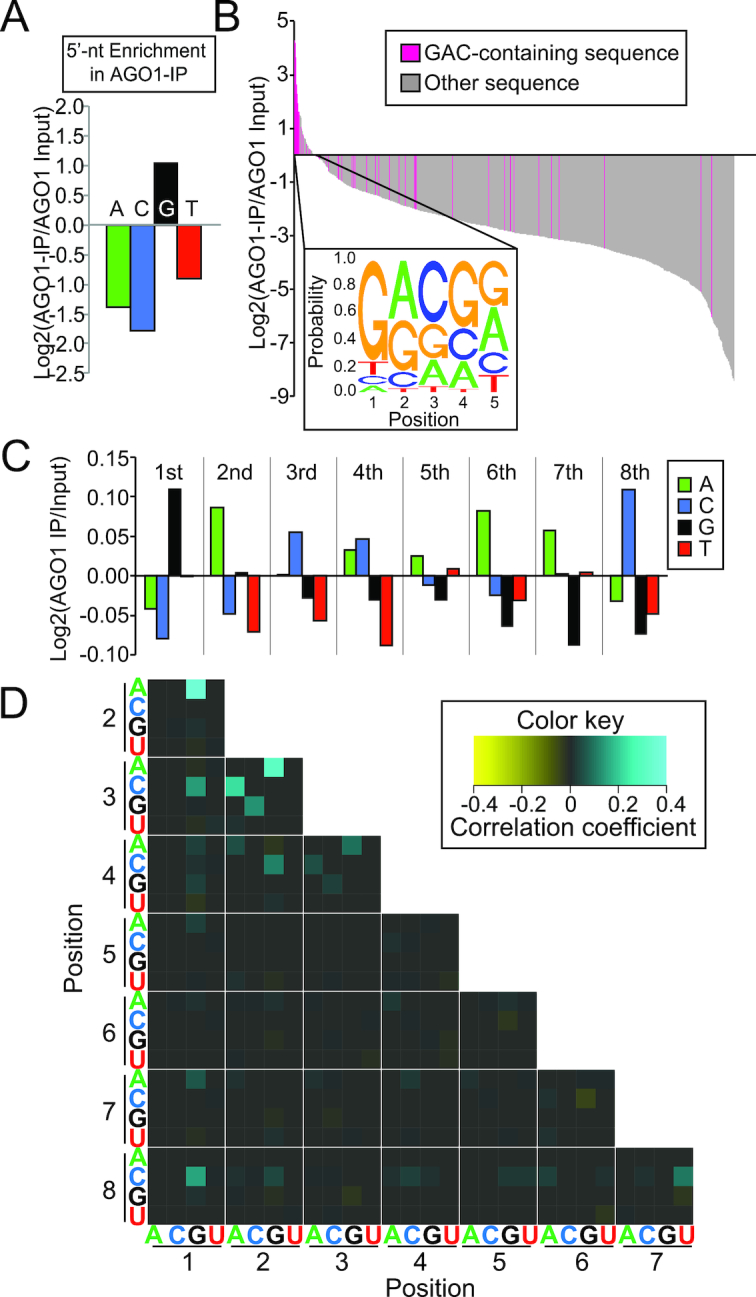
GAC enrichment in the AGO1-HISSA libraries. (**A**) Enrichment factors of the 5′-nucleotides in 5′-5N-AGO1-HISSA. The enrichment factor of each nucleotide was calculated by dividing the read count in the 5′-5N-AGO1-IP by that in the 5′-5N-AGO1-Input library for each nucleotide. Log_2_ transformed values are shown. 5′-G showed strong enrichment count in the 5’-5N-AGO1-IP library. (**B**) Summary of 5’-5N-HISSA results for AGO1. The chart format is the same as Figure 4C. In the bar chart, sequences with GAC are highlighted. (**C**) GAC enrichment revealed by the 8-nt randomization HISSA. The chart format is the same as Figure 4D. (**D**) Pairwise analysis supports GAC as a motif. The correlation coefficient between a given 2-nucleotide combination and AGO1 enrichment was calculated. Note that coefficient values were used only when statistically significant correlation was observed. To reduce the number of combinations, only four positions were considered at a time and repeated the analysis for the 70 4-nt windows to cover the entire 8 randomized nucleotides. Averages of coefficient factors were shown when significant coefficients were obtained in multiple 4-nt windows. Higher coefficients indicate better loading efficiencies of the 2-nt combinations to AGO1. Series of signals corresponding to the GAC motif starting at positions 1, 2 and 3 were observed.

Next, we analyzed AGO1-HISSA results. Similar to the AGO2 dataset, we calculated enrichment factors for individual sequences using normalized read counts for 5′-5N libraries. In this set, we were again able to detect almost all species (1019/1024 in Input and 937/1024 in IP with >99 reads; 1024/1024 in Input and 1022/1024 in IP with ≥1 read) (Supplementary Table S2). To our surprise, we did not find evidence for 5′U-enrichment by this analysis but detected enrichment of species starting with Gs (Figure 5A and Supplementary Figure S5).

It is possible that structured RNA molecules are more stable in the lysate due to their resistance to ribonucleases specific for ssRNAs. Their structures might explain the unexpected G enrichment at the 5′ end because Gs have the ability to form non-canonical G-U wobble pairs in addition to G-C pairs with three hydrogen bonds. We examined the distributions of predicted folding free energy values of the species found in the input and IP libraries. However, we observed no evidence that AGO1-IP enriches more highly structured molecules (Supplementary Figure S6). For 5′-5N libraries, oligo sequences seen in the AGO1-IP library were predicted to have lower probability to form structures than those in the input library, arguing against the possibility that AGO1 selects more highly structured short RNA molecules.

We next tested whether particular motifs were associated with enrichment or depletion in the AGO1-IP library, we looked for 3nt-motifs associated with the loading efficiency using Bind-n-seq. Among the 64 possible 3-nt motifs, GAC was strongly associated with higher enrichment in the AGO1 library (Supplementary Table S2). When we selected highly AGO1-enriched sequences, we observed frequent occurrence of GAC near the 5′ end (Figure 5B logo, Supplementary Figure S7A). In addition, sequences containing GAC at the second window (nucleotides 2–4) were also enriched in AGO1-IP HISSA. To test if the 5′G-enrichment in the AGO1-IP HISSA library could be solely explained by the strong enrichment of 5′GAC-containing reads, we excluded reads starting with 5′NAC and repeated the 5′-nucleotide enrichment analysis (Supplementary Figure S7B). We observed a reduction of 5′G-enrichment in both 5′-5N and 5′-8N HISSA libraries but G enrichment values remained positive, suggesting that 5′G is still preferred even in non-NAC contexts. This may be reflecting the fact that 5′G has positive interaction with particular nucleotides at positions 4–8 as seen in the pairwise analysis of 5′-8N library (Figure 5D).

Similar to AGO2-HISSA, we were not able to directly compare the abundance of each sequence for the 5′-8N libraries, therefore we analyzed the enrichment and depletion of the four nucleotides at each position after AGO1-IP compared to the input population. Consistent with the 5′-5N-HISSA results, we observed GAC enrichment at the first three nucleotides of the oligo (Figure 5C, Supplementary Table S2). To further test whether the enrichment of the three nucleotides occurred independently or the three nucleotides showed association with each other, we performed pairwise correlation analysis (6). We tested whether a given combination of two nucleotides showed statistically significant correlation with AGO1 enrichment or depletion (Figure 5D, See materials and methods). Indeed, G and A in the first two nucleotides exhibited clear positive effects on loading (Figure 5D). This was followed by enrichment of the combinations A-C and G-C at positions 2–3 and 1–3, respectively. We additionally observed weaker G–A–C association starting at positions 2 and 3, suggesting that the effects of GAC was stronger at the 5′ end, but the GAC motif does not have to be located at the 5′-end to influence loading at a detectable level.

Because enrichment of G-rich and GAC-containing sequences were highly unexpected, we considered the possibility that the small RNAs enriched in the Argonaute HISSA libraries may represent RNA oligos that were recognized as targets by endogenous small RNA species in the Argonaute complexes. If this is the case, C-rich and GTC-containing sequences are expected to be enriched in the AGO2- and AGO1-loaded endogenous small RNA populations respectively. We analyzed published small RNA libraries prepared with AGO1 and FLAG-tagged AGO2 complexes purified from S2 cells (9). However, Cs were not enriched in AGO2-loaded small RNAs and GTC motif was not observed in the seed region of the AGO1-loaded species, therefore excluding the possibility that these G-rich and GAC-containing sequences represent RNA oligos recognized as targets by endogenous small RNAs (Supplementary Figure S8).

The results supported the idea that the 5′-GAC sequence acts as a motif rather than reflecting preferred nucleotides at the three positions. The effects could be observed even when the GAC motif was present at positions 2–4 and 3–5, albeit to a lesser extent. This further supported the notion that the presence of the GAC motif promotes loading of the ssRNA species on AGO1.

### 5′G is preferred to 5′U for AGO1 loading in the context of 5′-NAC

**Figure 6.**
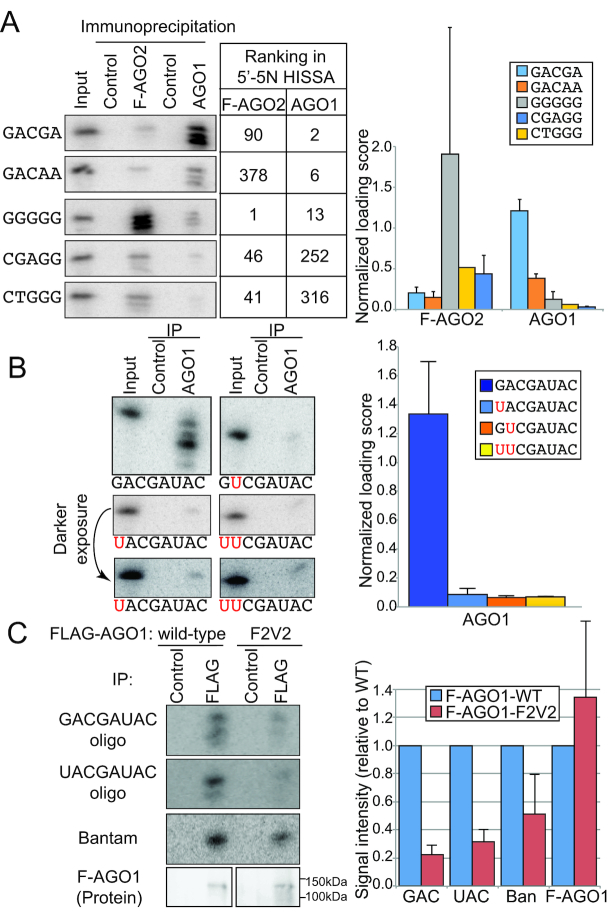
5′GAC is better than 5′U for AGO1 loading. (**A**) Validation of HISSA results using individually synthesized RNA oligos. To verify the 5′-5N-HISSA results, 5 representative RNA oligo nucleotides were individually synthesized. The rankings in 5′-5N-HISSA data for FLAG-AGO2 and AGO1 are shown in the table. The table is sorted according to the ranking in AGO1-HISSA. The chart on the left shows the *in vitro* loading results for each variant. Input is 0.01% of oligos introduced in an IP reaction. Loading scores were calculated by the following formula: (signal intensity in the Argonaute-IP lane) × 100/(signal intensity in the Input lane). The loading efficiency measured with the individual oligos confirms the accuracy of HISSA results. The right chart shows averages and standard deviations of the *in vitro* loading assay results (*N* = 2). (**B**) 5′ G within the GAC motif is preferred over 5′ U species. To test the importance of GAC, the 5′G was changed to 5′U (UACGA: left middle and lower panel) or the second A was changed to second U (GUCGA: right upper panel). Both of the variants showed lower AGO1 loading efficiency compared to the GAC variant. This was not due to additive effects of individual nucleotides, because changing the first two nucleotides did not further dampen the loading efficiency compared to the GUC and UAC variants (UUCGA: right middle and lower panels). The right chart shows averages and standard deviations of the *in vitro* loading assay results (*N* = 3). (**C**) *In vitro* loading reactions were done using lysates prepared from S2 cells transiently expressing the indicated wild-type or F2V2 mutant AGO1 tagged with FLAG at the N-terminus (46,61). For F2V2 mutant, the level of AGO1 protein was lower in the cell extract potentially due to the mechanism degrading empty AGO1 (Supplementary Figure S10A) (68,69). The effects appear to be post-transcriptional because both plasmids produced similar levels of AGO1 transcript (Supplementary Figure S10B). To account for the difference in the protein level, we used six times more materials for *in vitro* loading for the F2V2 mutant to obtain the amount of AGO1 protein equivalent to the FLAG-tagged wild-type AGO1 protein. The amounts of the precipitated proteins were assessed by Western blotting against FLAG-tag, using the protein samples immuno-precipitated from the same batches of lysates as the *in vitro* loading reactions under the same immunoprecipitation condition. Co-precipitated RNA oligos were detected by autoradiography. The quantified signals of Northern (*N* = 3) and Western blotting (*N* = 2) results were normalized by the values from the wild-type AGO1 immunoprecipitation lane.

Since HISSA involves PCR amplification, which often introduces errors to quantitative results, we wished to verify our results with a method that does not require PCR amplification. Therefore, we synthesized RNA oligos with sequences selected based on the ranking in HISSA results and used them for *in vitro* loading experiments (Figure 6A, See Supplementary Figure S9A for full gel images.). We chose five oligos that ranked in the range of 1st to 378th in AGO1 or AGO2 for the verification experiments. The quantified *in vitro* loading values showed a good correlation with the rankings of oligos in AGO1- and AGO2-IP libraries. Therefore, these experiments excluded the possibility that our HISSA results were strongly affected by PCR amplification or sequencing artifacts.

Having confirmed the accuracy, we were interested in verifying the role of GAC in enhancing ssRNA loading into AGO1. Even though AGO1 is known to prefer 5′U (15), changing the 5′G of the GAC-variant (5′UAC-variant) lessened the loading efficiency (Figure 6B lower left panel, Supplementary Figure S9B). We predicted that 5′G-mediated enhancement of AGO1 loading would be specifically observed when the following nucleotides are AC, since GAC occurs as a motif most strongly in the first to third positions (Figure 5D). To test this hypothesis, we made two additional variants having 5′U or 5′G followed by UC at the second and third positions (Figure 6B right panels, Supplementary Figure S9B). In the context of 5′NUC, 5′G was not more effective in enhancing AGO1 loading compared to 5′U.

Resistance of the binding between the RNA oligo and AGO1 protein to our relatively stringent detergent condition (0.1% SDS, 0.5% sodium deoxycholate, 0.5% NP40) suggested that the interactions are strong, as seen with properly loaded guide RNA molecules and Argonaute proteins (45). However, it was formally possible that the RNA oligos co-precipitated with AGO1 were not properly loaded in the guide RNA binding channel of AGO1 in our ssRNA loading experiments, and the binding may have been a mere artifact reflecting fortuitous contacts between the guide RNA and AGO1 molecules. To exclude this possibility, we made use of previously generated mutant AGO1 with point mutations in the MID domain that are expected to interfere with binding the guide RNA 5′ region (F2V2 mutant) (Supplementary Figure S10) (46,47). As expected, an endogenous miRNA Bantam was less strongly enriched by the immunopreciptated F2V2 mutant AGO1 compared with the wild-type AGO1 (Figure 6C). Under this condition, *in vitro* loading reaction yielded lower amounts of co-immunoprecipitated guide RNA with both model oligos harboring GAC or UAC at the 5′ end (Figure 6C and Supplementary Figure S9C). This indicated that the integrity of the MID domain is important for stable binding of RNA molecules introduced in *in vitro* reactions, providing evidence that RNA molecules co-precipitated with the AGO1 complex were properly loaded in the guide RNA binding cleft of AGO1.

All these results supported the conclusions of HISSA, and further confirmed the role of GAC in facilitating AGO1 loading in *in vitro* systems.

### Sequence bias in AGO1-bound endogenous sRNAs

We wondered if the preferred AGO1-loading of GAC-containing ssRNAs is reflected in endogenous sRNA populations bound by AGO1. To test this, we reanalyzed published small RNA sequencing libraries constructed with RNA extracted from the purified AGO1 complex as well as total RNA samples from fly heads and ovaries (15,48). Since the majority of AGO-loaded species are derived from small RNA duplexes produced by Dicer and the duplex structure has strong influence on loading efficiency, it is unlikely that the sequence preference can be observed with these known sRNA populations derived from duplexes. We hypothesized that the sequence preference in Argonaute loading could be more evident with the minor populations that were derived from non-hairpin ssRNA species.

**Figure 7.**
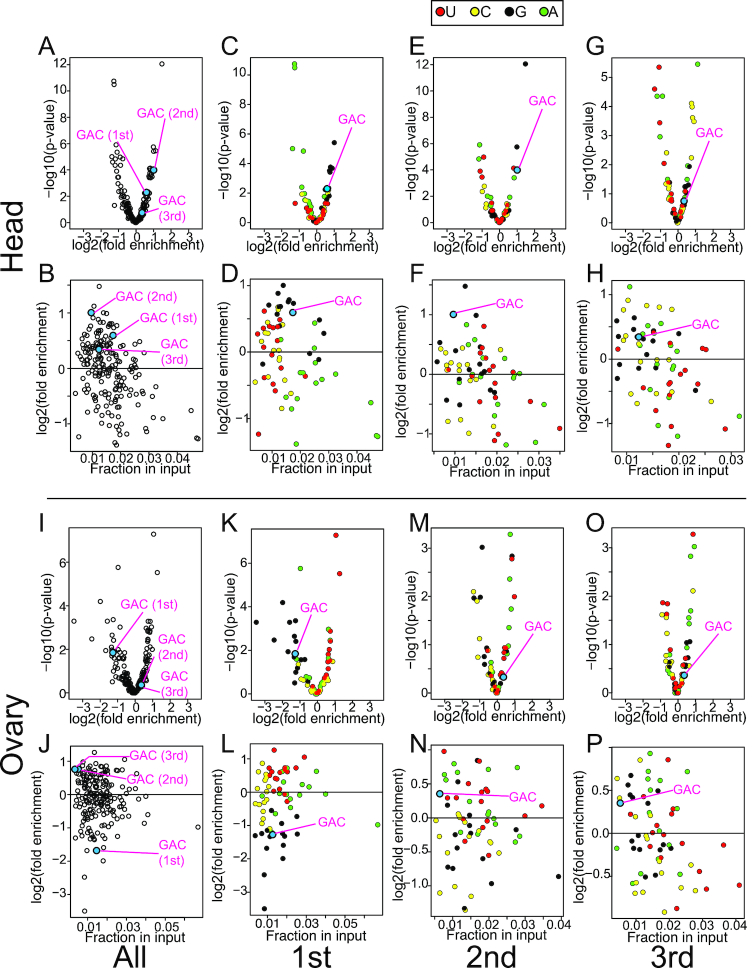
Sequence bias of AGO1-bound species *in vivo*. sRNAs derived from non-duplex origins were enriched by serial removal of known or potential duplex-derived species by the bioinformatics filters (Supplementary Figure S11) using previously published AGO1-IP and total RNA libraries from fly heads (15) and ovaries (48). The remaining reads were used for this analysis and the results for datasets using heads (**A**–**H**) and ovaries (**I**–**P**) are shown. We extracted sequences of 3nt windows starting from first, second and third nucleotides, and counted reads sharing the same 3-nt motifs. Fold enrichment values were calculated by dividing the percentage of reads containing the motif in the IP library by that in the input library. The charts show results for all three 3nt-windows combined (A, B, I and J), first window (C, D, K and L), second window (E, F, M and N) and third window (G, H, O and P). Upper panels for each of the head and ovary sections show volcano plots with log2 of Fold enrichment and –log_10_ of Fisher's exact *P*-value on the x and y axis, respectively. In the bottom charts, log_2_ of Fold enrichment was plotted against percentage of reads containing the motif in the input library. In panels C–H and K–P, the first nucleotides of each window was color coded with A, C, G and U represented by green, yellow, black and red dots, except for reads with GAC that were represented by the light blue dot.

To obtain reads that represent ssRNA fragments derived from annotated mRNAs, we applied a set of bioinformatics filters to the library data to exclude reads derived from duplexes as much as possible (Supplementary Figure S11; see Materials and Methods). Because of the abundant genic piRNAs produced at least in somatic tissues of the ovary (49,50), we needed an additional filter to exclude them (32). Because of this complexity in the ovary, we note that the ovary dataset may not be as reliable as the head dataset. This yielded 1000–9000 reads uniquely mapped to mRNAs (Supplementary Table S3). We extracted 3-nt sequences starting at first, second and third positions counting from the 5′ end of the read to examine whether each of the 3-nt motifs was enriched or depleted in the AGO1-IP library compared to the input library (Figure 7).

In the head libraries, we observed slight 5′G enrichment (Figure 7C and D), and we did not see an obvious enrichment or depletion of a particular base at the second and third positions (Figure 7E–H). 5′G enrichment was not seen in the ovary dataset (Figure 7K and L). The head results were unexpected because 5′U is believed to be preferred for AGO1 binding (15,18). The results indicated that 5′U-enrichment is not a universal phenomenon seen with sRNA populations co-precipitated with AGO1, as observed in HISSA. 5′G enrichment was also seen with non-5′-NAC context, suggesting that 5′G was generally preferred for AGO1 loading under this condition (Supplementary Figure S12A).

In the first 3-nt window in the head dataset, 5′-GAC was significantly enriched in the AGO1-IP library (1.5-fold enrichment, Fisher's exact test *P* = *0.005*). Again, consistent with the HISSA results, we observed enrichment of GAC at the second position, whose enrichment factor ranked second among the 64 sequences (2.0-fold enrichment, Fisher's exact test *P* = 1 × 10^−4^) while enrichment was much weaker at position 3 (1.3-fold enrichment, Fisher's exact test *P* = 0.18, Supplementary Figure S12B). In ovaries, 5′-GAC was depleted (2.4-fold depletion, Fisher's exact test *P* = 0.01), and no significant enrichment was seen at positions 2 and 3 (1.3-fold enrichment, Fisher's exact test *P* > 0.4 at both positions). This suggests that the effects may be context-dependent, perhaps with tissue specific effects.

These results suggested that the sequence preference in ssRNA loading into AGO1 might influence the compositions of AGO1-bound species derived from ssRNAs *in vivo* at least under certain conditions.

## DISCUSSION

### Utility of HISSA

We developed HISSA as a tool to study roles of nucleotide sequences in ssRNA loading into Argonautes. HISSA uses splinted-ligation for sequence specific recovery of introduced oligos and exclusion of endogenous RNAs from the library (Figure 3, Supplementary Figure S4). Despite the low amount of the introduced oligos (∼0.75 fmol in ∼0.20 nmol of endogenous small RNA), we were able to construct libraries that were comprised mostly of the introduced oligos (Figure 3B). The use of HISSA is not limited only to Argonautes but it can be a powerful tool to study binding specificity of other RNA binding proteins. HISSA is useful when the protein sample contains large quantities of endogenous RNA species. Even when the RNA binding proteins are produced and isolated as recombinant proteins, protein samples often contain RNAs derived from host cells in which the recombinant proteins were expressed, depending on the purification methods (51). Those contaminants could be included in the RNAseq library with conventional library construction procedures and may eventually affect the analysis results. Due to the specificity of splinted-ligation, HISSA can amplify minute amounts of specific RNA species in the presence of a large amount of endogenous RNAs with diverse sequences and provides flexibility in the design of experiments. On the other hand, HISSA requires a constant region that serves as a bridge-oligo binding site for splinted-ligation, and RNA oligo sequences could be only partially randomized. Therefore, HISSA should be considered as an alternative to standard high-throughput methods such as Bind-n-Seq (42,43), when the presence of endogenous RNA species in the samples is expected to interfere with the experiments.

Oligonucleotide-based therapeutics is an emerging area, and some of the attempts have already produced successful results with official approvals as drugs by agencies (52). With some modifications in the experimental design, the HISSA platform may also be useful for analyzing *in vivo* kinetics and distribution of oligonucleotides. Sequence-specific 3′-ligation and sequencing-based detection and quantification would be a powerful tool when detection of full length oligonucleotides with high sequence specificity is required, because it is difficult to achieve specificity at the single-nucleotide level with qPCR-based detection methods. Furthermore, HISSA would allow simultaneous detection of various oligonucleotides from biological samples containing endogenously derived RNA or DNA, and could be an avenue to rapid screening for sequences that show ideal degradation or distribution patterns. Furthermore, *in vivo* stability and tissue distribution of oligonucleotides with various chemical modifications could be simultaneously tested by introducing barcode sequences that are associated with chemical modifications introduced to the oligonucleotide unless the chemical modifications block the reverse transcription reaction.

With its flexibility, HISSA has potential as a platform of various biochemical and biomedical engineering studies.

### Mechanisms of ssRNA loading

While the mechanism of small RNA duplex loading has been extensively studied, how ssRNAs are loaded into Argonautes is not known (53). Results using recombinant proteins suggest that spontaneous loading of ssRNAs can occur in a manner independent of chaperones and dsRNA-binding protein co-factors in *in vitro* experiments (9,54). These results do not exclude the possibility that other loading factors might be involved to specifically facilitate binding of ssRNAs to Argonautes.

What is the molecular mechanism that is responsible for the sequence specificity in ssRNA loading? Our results suggest that degradation rates are not a major determinant of loading efficiency (Figure 1C). Some unknown cofactor with some sequence specificity might be involved in the preferential loading of particular ssRNA species. However, a potentially more interesting possibility is that Argonautes may have their own sequence preferences. The past structural studies did not discover extensive interactions between the Argonaute protein surfaces and bases of loaded small RNAs, but this notion was formed based on studies using a small number of model RNA species (55,56). The possibility that a subset of guide RNAs with certain sequences may form contacts between RNA bases and the surfaces of Argonautes cannot be excluded. Interestingly, a recent study demonstrated that sequences of the guide RNAs affect the behaviors of human Argonaute proteins against perfectly complementary targets (57). Human Ago3 is generally more sensitive to the guide RNA sequence than human Ago2 when it cleaves perfectly complementary targets, explaining why this Argonaute was long believed to be a non-slicer. Future studies should aim to test whether Argonaute proteins by themselves show sequence specificity for guide RNAs.

### Potential evolutionary origin of sequence preferences in Argonaute loading

An emerging concept is that the ancestral Argonaute gene arose as a genome-defense effector that silences foreign nucleic acids in prokaryotes (58). Argonautes also play roles in genome defense in the majority, if not all, of eukaryotes, while some subsets of Argonaute proteins have acquired new roles in gene regulation during evolution (3).

In unicellular organisms, ssRNA loading is more commonly observed. In fission yeast, Ago1 binds ssRNA fragments from bidirectionally transcribed regions and the loaded ssRNA fragments, known as priRNAs (primal RNAs), trigger amplification of siRNAs that initiate heterochromatin formation at target loci (59). The prokaryote *Rhodobacter* encodes an Argonaute that utilizes guide RNA to cleave target DNA as a natural defense system against phages (60–62), but how the *Rhodobacter* Ago preferentially load guide RNA from phages and plasmids is not known. In these cases, ssRNA loading was assumed to be sequence non-specific except for the 5′ nucleotide preference, but whether loading into yeast Ago1 and *Rhodobacter* Ago is truly sequence-independent remains to be tested. Furthermore, structural and biochemical studies showed that *Rhodobacter* Ago releases guide RNAs in the presence of target DNAs with mismatches at particular positions (62). This effect might contribute to seeming loading preference by getting rid of sRNAs with particular sequences from the Rhodobacter Ago. Interestingly, similar unloading mechanisms may be conserved in mammals (63,64). If there is any specificity in Argonaute loading or unloading in these organisms, it might contribute to the selective establishment of silent chromatin or preferential targeting of foreign DNA elements. HISSA using the materials from these organisms would provide an answer to this question.

Recently, Dicer-like proteins from *Paramecium* were demonstrated to unexpectedly have sequence specificity, and the sequence selective dicing was important for recognition of transposon-derived sequences (65). Therefore, the sequence specificity may emerge as a key characteristic of the genome defense factors when more evidence for sequence specific actions of those factors accumulates. Due to the low abundance of ssRNA-derived species in mature Argonaute complexes, we were unable to test whether the sequence preferences by *Drosophila* Argonautes are reflected in the target repertoire. However, such sequence preferences in ssRNA-loading may be more important in certain organisms. In fission yeast, ssRNA-derived sRNAs trigger amplification of sRNAs from target RNAs and in such cases, sequence specificity of ssRNA loading can more strongly influence the entire repertoire of Argonaute-loaded species as well as their targets (59,66).

### Sequence preference in ssRNA loading and its potential in RNAi technology

Researchers demonstrated that ssRNA-mediated RNAi has strengths over conventional duplex-mediated RNA methods especially for *in vivo* delivery including potential therapeutic applications (16,17,67). However, it has its own weaknesses, because the single-stranded nucleic acids can act as ‘antisense oligos’ without binding to Argonautes rather than functioning as guide RNAs for Argonautes. In fact, changes in the splicing pattern of Ataxin-3 (ATX-3) mRNA was observed by introduction of ss-oligonucleotides against the expanded CAG repeat seen in ATX-3 mRNA in fibroblasts derived from a Machado–Joseph Disease patient, in addition to the expected silencing of the target gene via the RNAi pathway (67). Although the dual function of ss-oligonucleotides may be beneficial in certain cases, it may cause unfavorable consequences in other cases. Therefore, it would be best if we could design ssRNA oligos that show ideal loading efficiency into Argonautes to minimize unfavorable effects and maximize the efficacy.

It should be noted that Ago2 is the catalytic engine of RISC in mammals, and *Drosophila* AGO1 is the direct counterpart of human Ago2. We found that ssRNA species were loaded onto human Ago2 at distinct rates, suggesting that ssRNA loading to human Ago2 also conforms to sequence specificity (Figure 2). If human Argonaute proteins also exhibits sequence specificity, Argonaute HISSA using human materials may offer important information for better ss-siRNA designs.

For newer generations of RNAi technologies to emerge, these fundamental questions regarding biochemical properties of Argonautes need to be answered. Future studies using human materials to clarify ssRNA loading specificity may pave the way to the next innovations in RNA silencing technologies.

## DATA AVAILABILITY

The small RNA library data produced for this study are deposited at NCBI SRA under PRJNA478767 (SRR7458819 to SRR7458826).

## Supplementary Material

Supplementary Data
