# Supplementary material for: Hidden sequence specificity in loading of single-stranded RNAs onto Drosophila Argonautes
Source: Nucleic Acids Res. 2018 Dec 27;47(6):3101–16. doi: 10.1093/nar/gky1300 (PMC6451100; doi:10.1093/nar/gky1300)
Supplement: Supplementary Data [file gky1300_supplemental_files.zip › Merged_Supplementary.pdf]

# Hidden sequence specificity in loading of single-stranded RNAs onto *Drosophila* Argonautes

Eling Goh[1,2] and Katsutomo Okamura[1,2]

[1]Temasek Life Sciences Laboratory, 1 Research Link, National University of Singapore 117604, Singapore

[2]School of Biological Sciences, Nanyang Technological University
60 Nanyang Drive, Singapore 639798, Singapore

## Supplementary Information
Supplementary Figures S1-S12
Legends of Supplementary Tables S1-S4

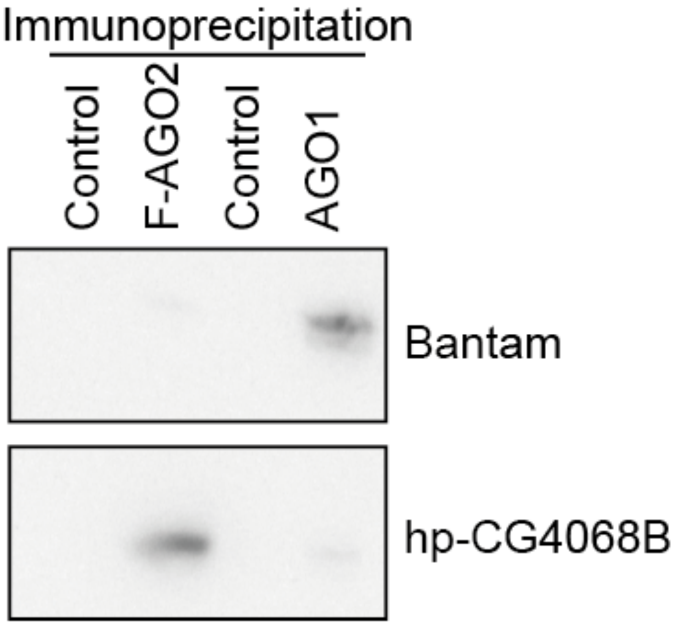


**Figure S1. Specificity of the Argonaute complex purification.** Membranes used for in vitro loading were probed for an endogenous miRNA (Bantam) and an endogenous siRNA (hp-CG4068B). As expected, the siRNA and the miRNA were enriched in FLAG-AGO2 and AGO1 complexes, respectively. Shown are results using membranes for in vitro loading of mir-34 loop short 5’ swap, and two different replicate membranes were used for this figure.

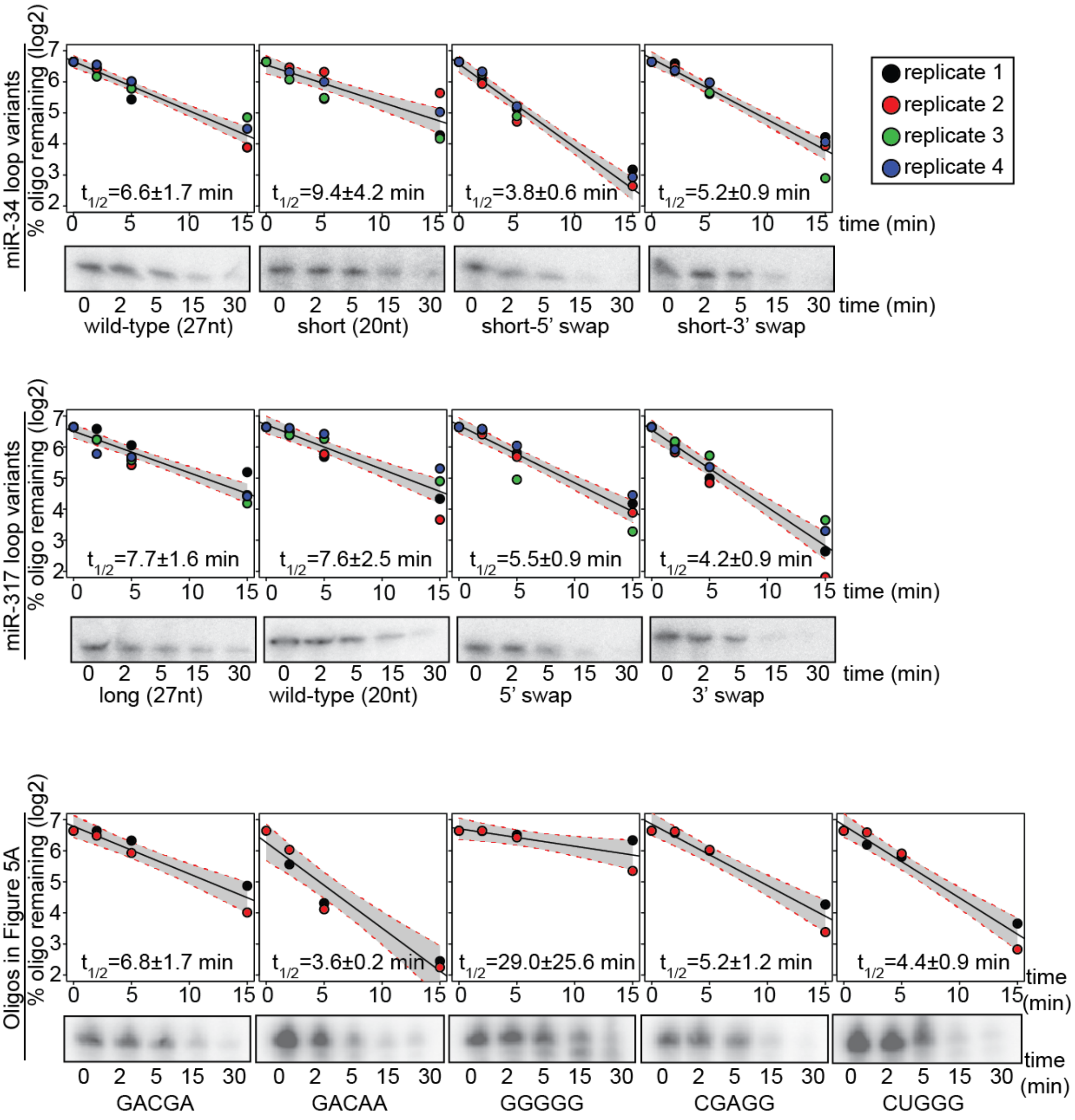


**Figure S2. Gel pictures of degradation assays used to obtain half-lives of oligos in Figure 1C**. 5'-$^{32}$P-labeled oligos were added to lysate and RNA was extracted directly from 10µl of the reaction at five time points – 0, 2, 5, 15 and 30 minutes. Samples were loaded into 15% denaturing gel. The reading from each time point was normalized to the reading at 0 min and expressed in the percentage. $\log_2$ of normalized % values were plotted against time (in minutes) in the scatter plot. The readings with the incubation time of 30 minutes were excluded because signal intensities were generally very low. The half-life ($t_{1/2}$) of each oligo sequence was calculated using the best-fit line equation for each replicate, and then the average and standard deviation were calculated. For the line chart, all replicates were considered together to draw the regression line and determine the 95% confidence intervals (grey).

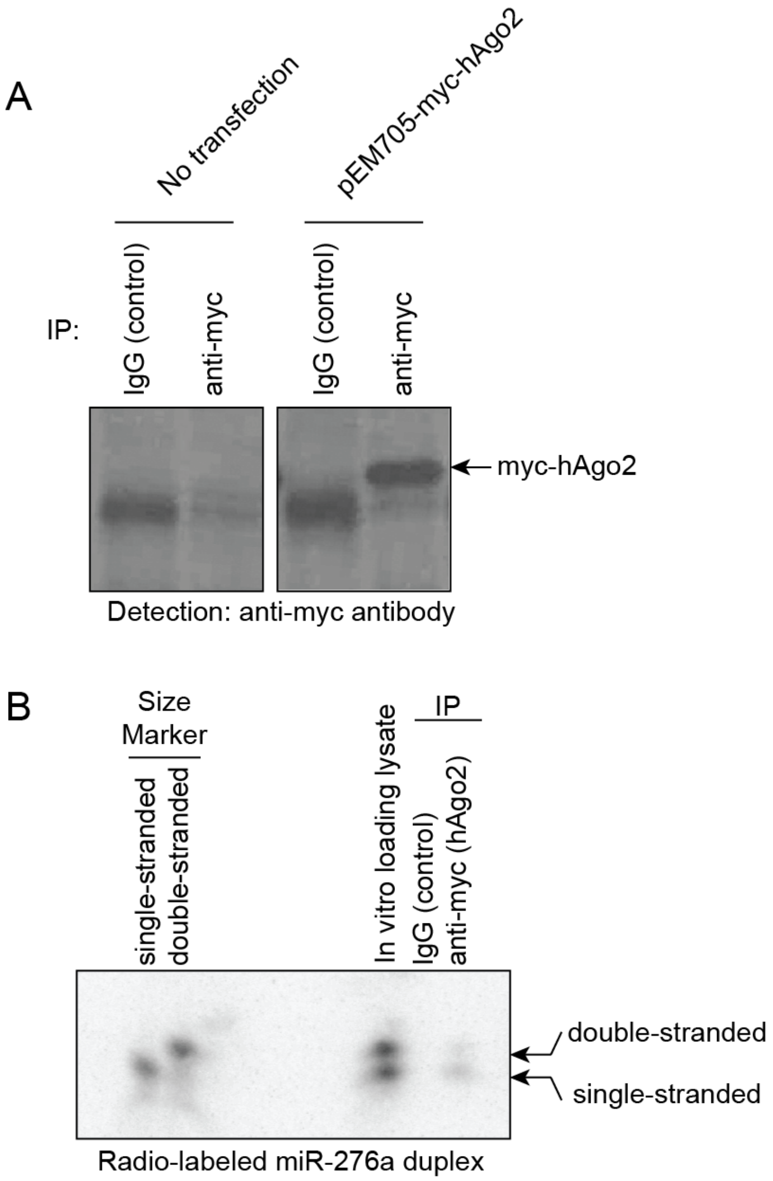


**Figure S3. Establishment of an in vitro Ago2 loading system in human cell lysates.** Lysates were prepared from HEK293T cells expressing myc-tagged Ago2. (A) Protein expression was detected by Western blotting using anti-myc antibody after performing immunoprecipitation with anti-myc-tag antibody from lysates of parental HEK293T-A2 cells or HEK293T-A2 cells stably transfected with pEM705-myc-hAgo2. (B) A synthetic 5'-$^{32}$P-labeled small RNA duplex corresponding to the sequence of *Drosophila mir-276a* was incubated in the cell lysate and the myc-Ago2 complex was immunopurified from the reaction. RNA extracted from the purified complex was analyzed on a 15% Native Acrylamide gel. Note that the Ago2 complex enriched the unwound species suggesting that the lysate supports loading of small RNA duplexes.

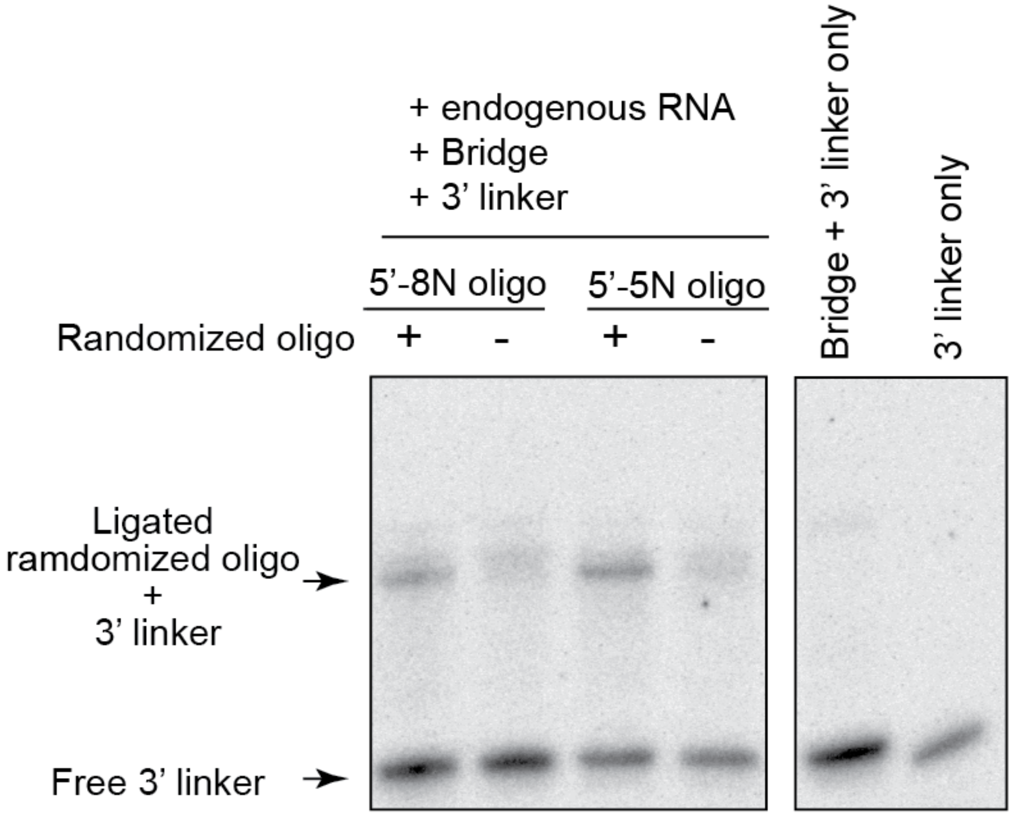


**Figure S4. Addition of 3’ linker to the 3’end of introduced oligo by splinted ligation.** Both splinted ligation bridges were designed to have 8nt complementarity to the 5’ end of 3’ linker while differed in the number of base-pairings with the 3’ end of randomized oligo. The bridges had 12nt and 15nt complementarity to the 3’ end of 8nt (first and second lanes) and 5nt randomized oligos (third and fourth lanes), respectively. To test the bridge specificity used for each randomized oligo, two reactions were set up. One contained both endogenous small RNAs and randomized oligo while the other contained only endogenous small RNAs. The specificity of each bridge is reflected by the ratio of band intensities; (endogenous small RNA + randomized oligo):(endogenous small RNA).

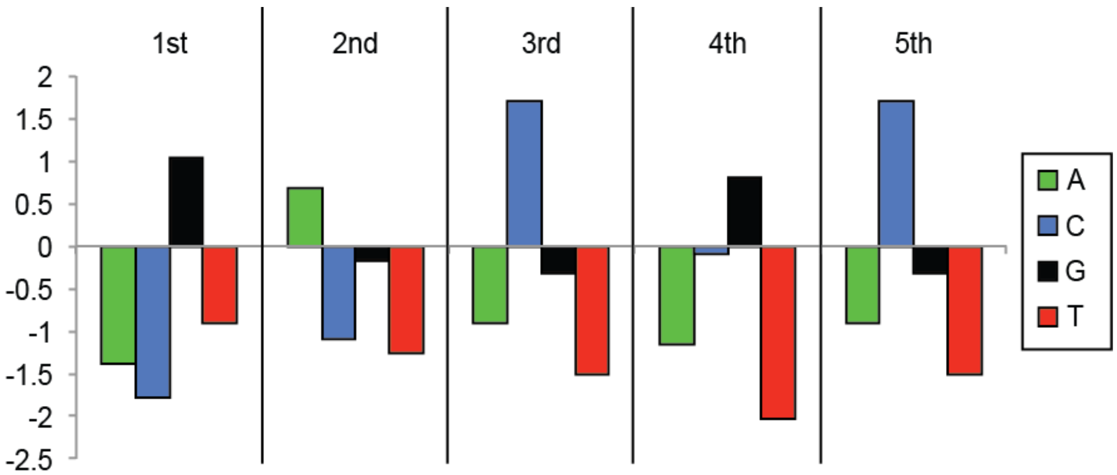


**Figure S5. Nucleotide enrichment for the 5 randomized positions in 5'-5N HISSA showed 5'G instead of 5'U preferred in AGO1 loading.** The percentage of reads having each nucleotide (A, T, G, C) at each position ($1^{st}$-$5^{th}$) was calculated, and the ratio of the percentages between the AGO1-IP and the corresponding input libraries were calculated and log2 transformed. Note that the data for the $1^{st}$ position are the same as Figure 5A.

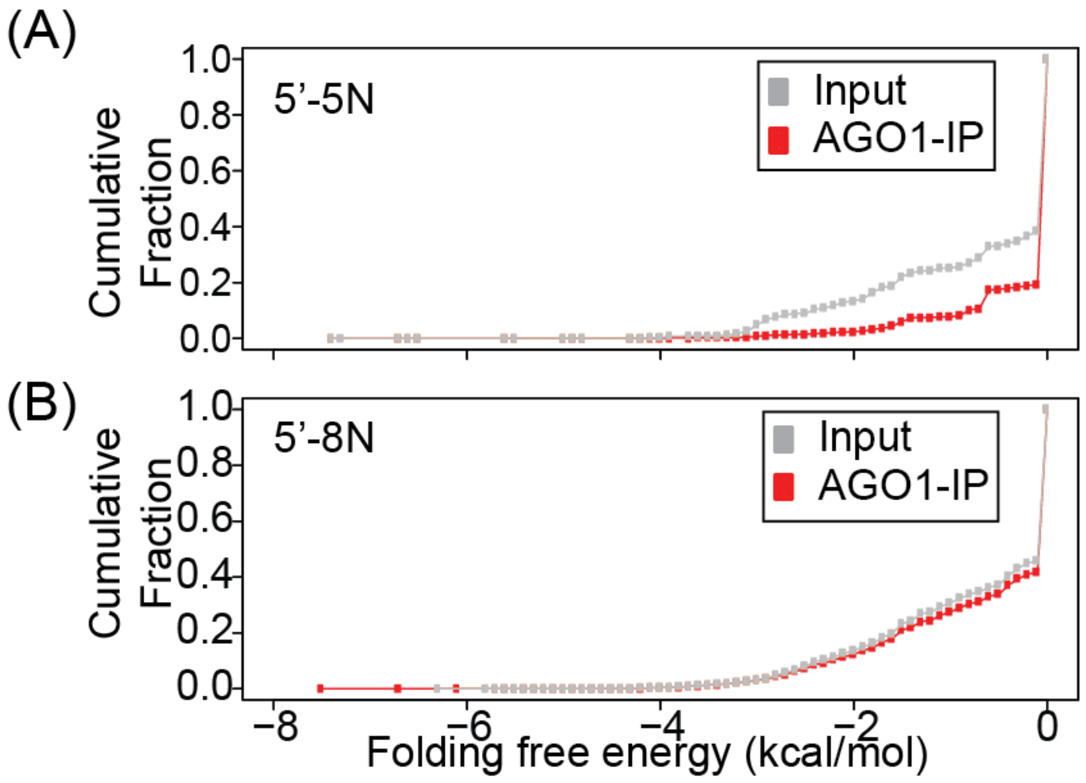


**Figure S6. Distributions of predicted folding free energy of sequences in 5’-5N (A) and 5’-8N (B) libraries.** Folding free energy was calculated by ViennaRNA (69) using the entire RNA oligo sequences including the randomized and backbone regions. The red and gray lines indicate cumulative distributions in the input and AGO1-IP libraries, respectively.

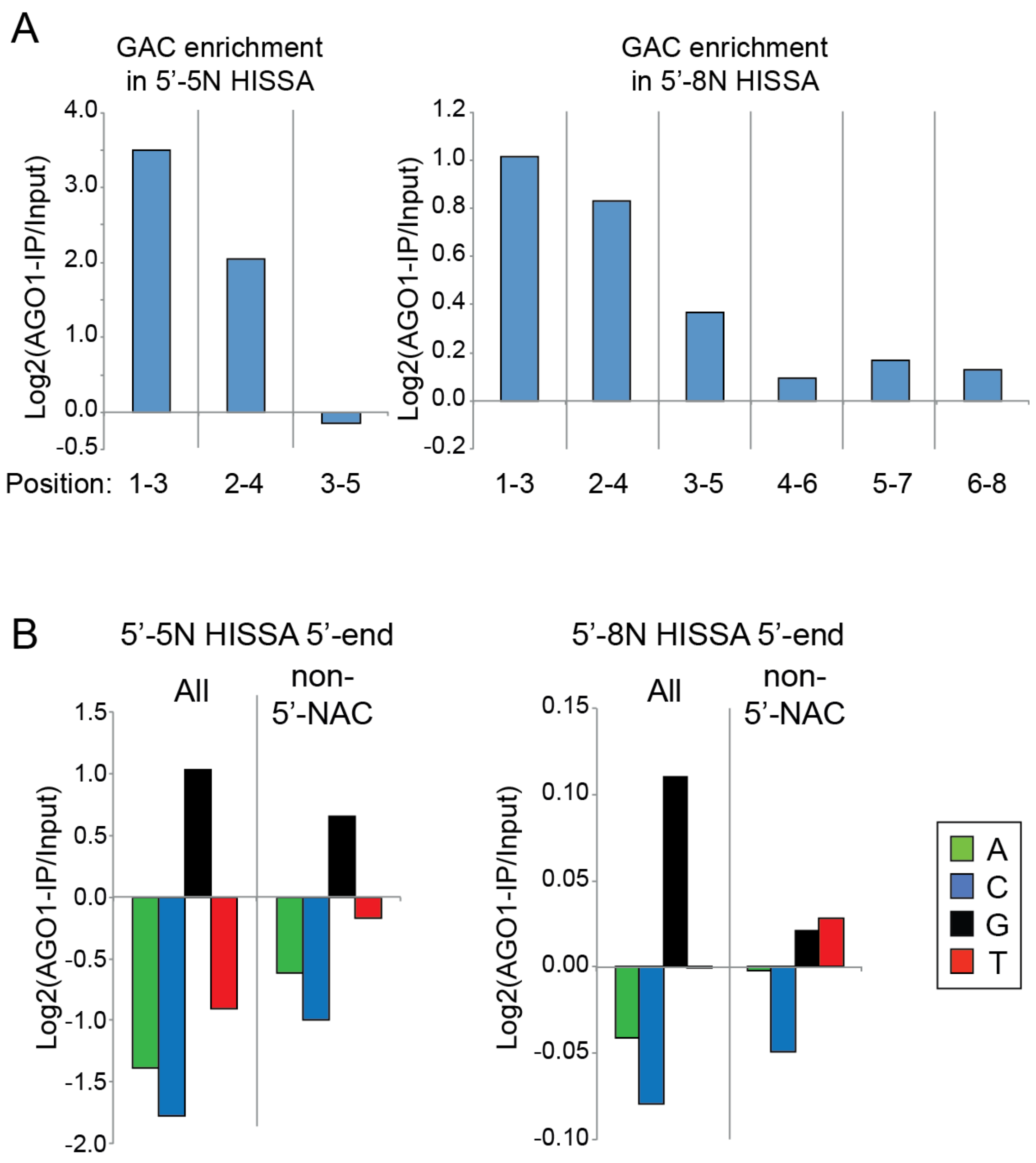


**Figure S7. Positional GAC-enrichment and 5'-nucleotide preference in the non-NAC context in AGO1-IP HISSA libraries.** (A) Reads containing the GAC motif at each position in the AGO1-IP and the corresponding Input HISSA libraries were counted, and the enrichment factors were calculated by dividing the fraction of GAC-containing reads in the total RNA oligo population in AGO1-IP library by that in the Input library. The Y-axis shows log2 of the ratios, and the results for 5'-5N (left) and 5'-8N HISSA (right) are shown. (B) 5'-nucleotide analysis was done using all reads or the population excluding reads that contain "AC" at position 2-3.

A. Logo of AGO2-loaded endogenous small RNAs

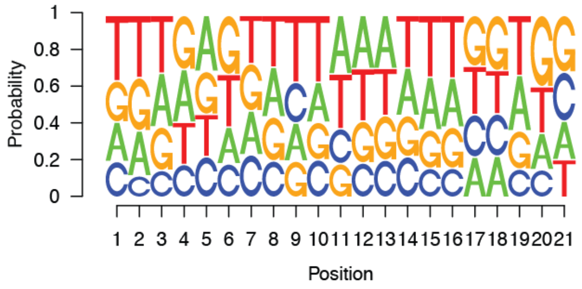


B. AGO1-loaded endogenous small RNAs

| Sequence | % |
|---|---|
| TGAGATCATTTTGAAAGCTGATT | 19.065 |
| TGAGATCATTTTGAAAGCTGAT | 9.9942 |
| TGGACGGAGAACTGATAAGGGC | 9.12792 |
| TATCACAGCCAGCTTTGATGAGCT | 6.64727 |
| TGGACGGAGAACTGATAAGGG | 4.30184 |
| TAATACTGTCAGGTAAAGATGTC | 2.74125 |
| TCACAGCCAGCTTTGATGAGCT | 2.19858 |
| CGTACGGTTTAAACTTCGA | 1.88285 |
| TATCACAGCCAGCTTTGAGGAGCG | 1.85148 |
| TATCACAGCCAGCTTTGAGGAG | 1.83159 |
| TCACAGCCAGCTTTGATGAGCTA | 1.16185 |
| TATCACAGCCATTTTGACGAGTT | 1.12005 |
| TGGCAGTGTGGTTAGCTGGTTGTG | 1.05142 |
| TATCACAGCCAGCTTTGAGGAGC | 0.896644 |
| CCGGTTTTCGATTTGGTTTGACT | 0.766804 |
| TGGACGGAGAACTGATAAGG | 0.693028 |
| TATCACAGCCATTTTGACGAGT | 0.691918 |
| TAGCTGCCTTGTGAAGGGCTT | 0.664809 |
| CATCACAGTCTGAGTTCTTGC | 0.613662 |
| ACGTACGCGGAATACTTCGATT | 0.437285 |
| TGGCAGTGTGGTTAGCTGGTTG | 0.375728 |
| TGAGATCATTTTGAAAGCTGA | 0.363549 |
| TAATACTGTCAGGTAAAGATGT | 0.316671 |
| TAGCACCATGAGATTCAGCTC | 0.259938 |
| TATCACAGCCAGCTTTGAGGAGCG | 0.250404 |
| TCTTTGGTGATTTTAGCTGTATG | 0.249341 |
| AGCGAGGTATAGAGTTCCTACG | 0.241704 |
| AGCAAGCTGACCCTGAAGTTCAT | 0.241667 |
| TGGCAGTGTGGTTAGCTGGTT | 0.218201 |
| TATCACAGCCAGCTTTGATGAGCT | 0.217023 |
| GTAGAGGAGATGGCGCAGGG | 0.213575 |
| TCAGGTACCTGAAGTAGCGCGCG | 0.192808 |
| CATCACAGTCTGAGTTCTTGCT | 0.18395 |
| TGGACGGAGAACTGATAAGGGCT | 0.167225 |
| TGGACGGAGAACTGATAATGGC | 0.14872 |
| CATCTTACCGGGCAGCATTAGA | 0.147777 |
| TGAGATCATTTTGAAAGCTGATC | 0.136249 |
| TATCACAGCCAGCTTTGATGAG | 0.132399 |
| TGAGATCATTTTGAAAGCTGATTT | 0.129666 |
| TAAATGCACTATCTGGTACGACA | 0.129111 |
| TGGACGGAGAACTGATAAGGGT | 0.121949 |
| TGGACGGAGAACTGATAAGGGCC | 0.121611 |
| TGGCAGTGTGGTTAGCTGGTTGT | 0.121477 |
| GTGCATTGTAGTCGCATTGTC | 0.108395 |
| CATCACAGTCTGAGTTCTTGCTT | 0.105575 |
| CATCACAGTCTGAGTTCTTG | 0.103882 |
| TCACAGCCAGCTTTGATGAGCTT | 0.100758 |

Seed

**Figure S8. Nucleotide enrichment and distribution of the GTC motif in endogenous sRNA populations in the AGO2- and AGO1-complexes.** Endogenous sRNA populations co-precipitated with FLAG-AGO2 and AGO1 complexes were analyzed using published libraries (9), and top ~550,000 and 47 abundant sequences in FLAG-AGO2- and AGO1-IP sRNA libraries were used

respectively. These sequences represent ~70% of each of the sRNA populations. (A) Sequence Logo was drawn with the top 70% AGO2-loaded endogenous sRNAs. C is generally underrepresented in AGO2-loaded sRNA populations. One would expect the opposite trend if the enrichment of G-rich sequences in the AGO2-HISSA libraries was a result of enrichment of RNA oligos co-precipitated with AGO2 as targets of endogenous sRNA species. (B) Distribution of the GTC-motif in AGO1-loaded sRNA populations. Sequences and their percentages in the AGO1-IP library are shown in the table. The GTC motif that is complementary to the GAC sequence is highlighted in red. 7 Sequences contained the GTC motif but none of them had the motif in the seed region ($2^{nd}$ -$7^{th}$ of the sRNA), arguing against the possibility that the enrichment of GAC-containing reads in the AGO1-HISSA library was a result of accumulation of GAC-containing RNA molecules as targets of endogenous sRNA species.

## A. Images used for Figure 6A

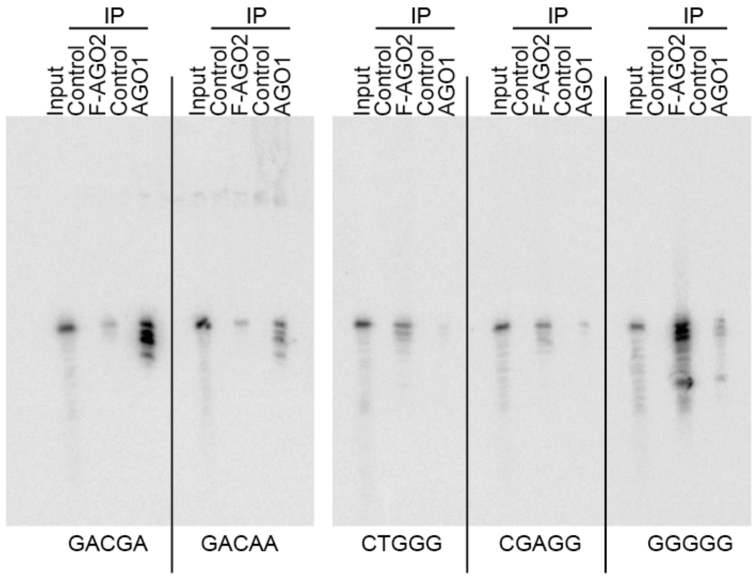


## B. Images used for Figure 6B

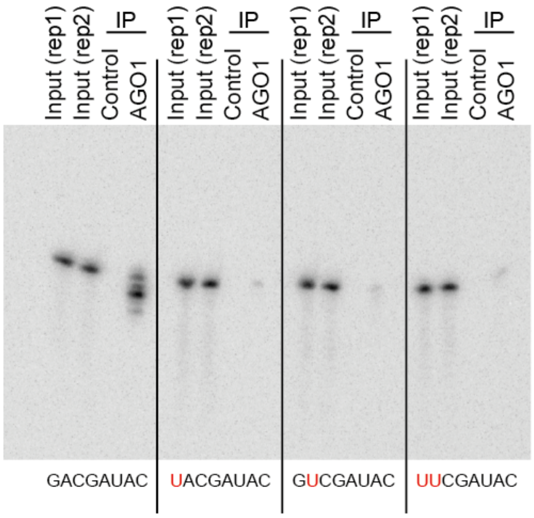


## C. Images used for Figure 6C

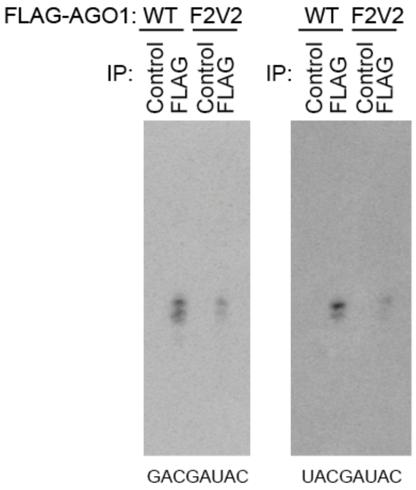


**Figure S9. Full images of gel pictures used for Figure 6.** Potential trimmed species were observed in some of the immunoprecipitated materials, but their identities were not known.

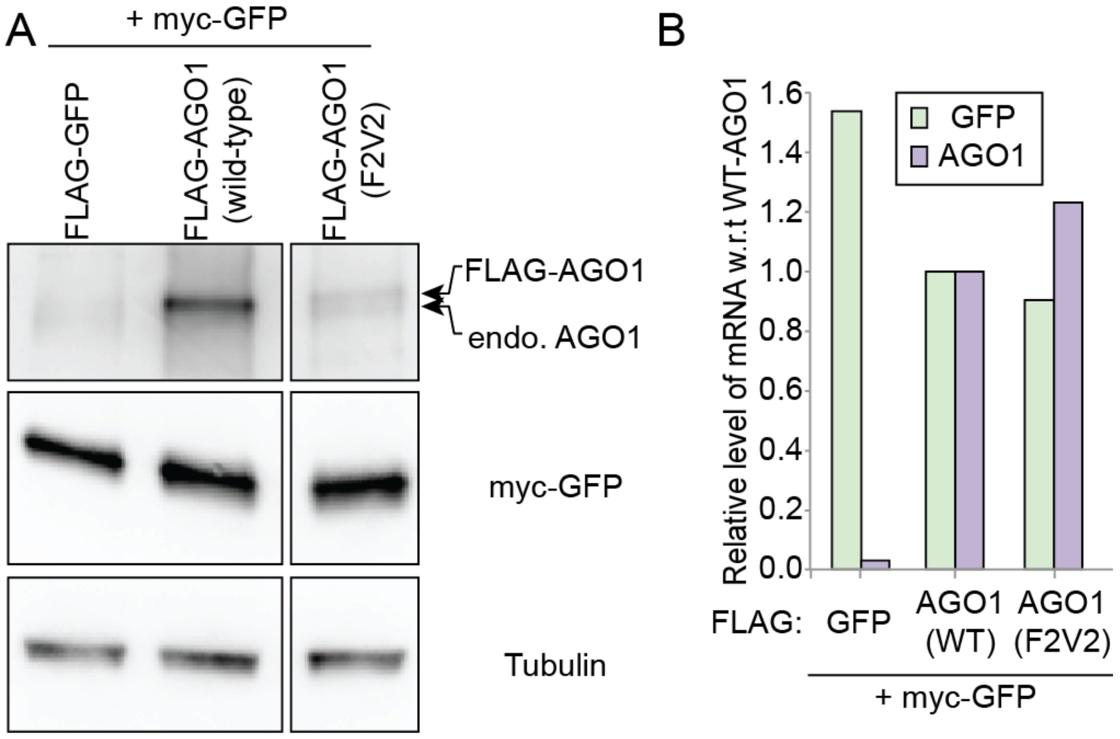


**Figure S10. Levels of FLAG-tagged AGO1 proteins.** (A) FLAG-tagged wild-type AGO1 or MID domain mutant AGO1 (F2V2) were expressed in S2-R+ cells and detected by anti-AGO1 antibody. Cells were co-transfected with a myc-GFP plasmid, and myc-GFP was detected by myc antibody to check the transfection efficiency. Similar signal intensities were observed in the three lanes. Alpha-tubulin was used as a loading control. The level of AGO1 protein was much lower when the mutant construct was used. Panels were cropped from the same gel pictures. (B) GFP and AGO1 transcript levels measured by qRT-PCR. Values were normalized to the internal control gene RpL32 and then the normalized values were further normalized to that in samples transfected with wild-type FLAG-AGO1. AGO1 transcript levels were similar between wild-type and F2V2 mutant despite the striking difference in the protein level.

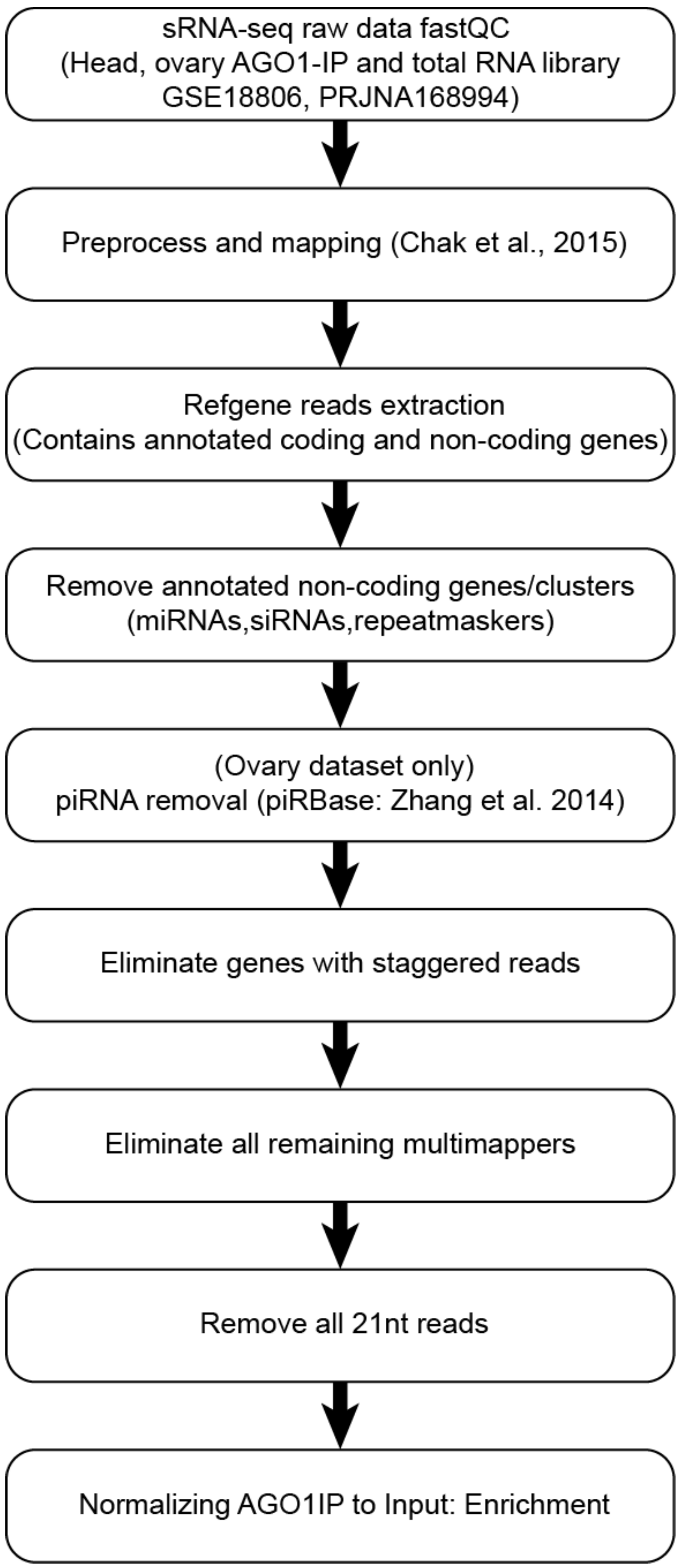


**Figure S11. Bioinformatics filters applied to the AGO1-IP and associated input libraries for mRNA fragment analysis.** Detailed information of the individual filters can be found in Materials and Methods.

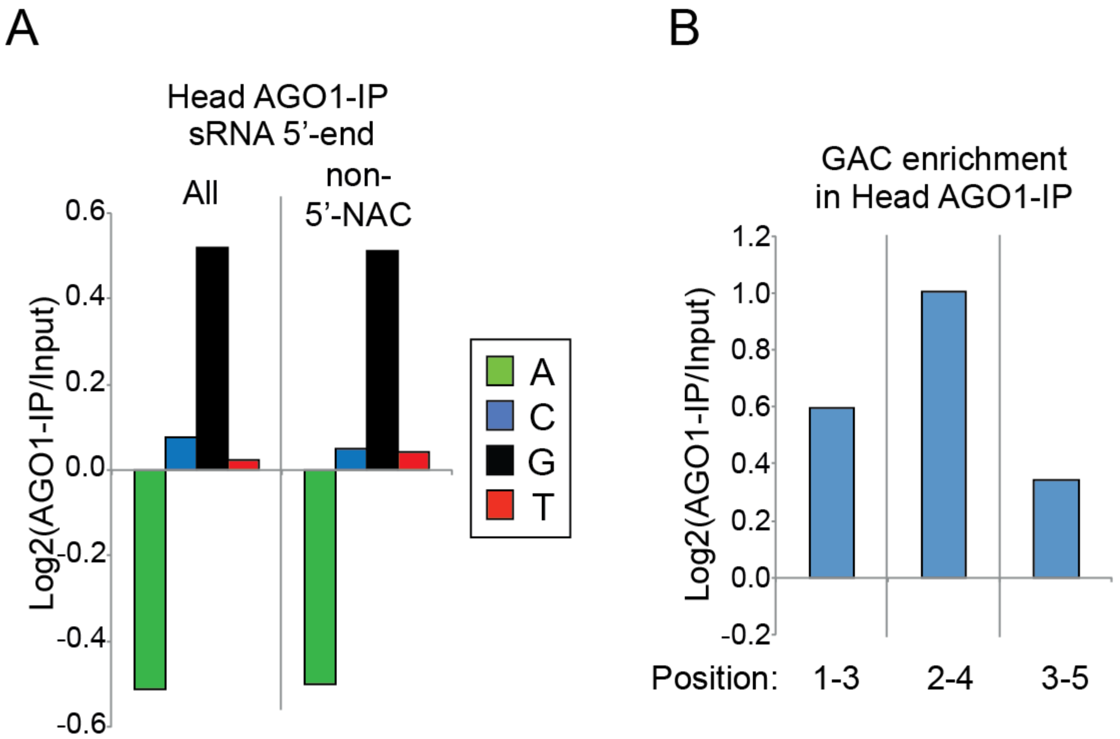


**Figure S12. Positional GAC-enrichment and 5'-nucleotide preference in the non-NAC context in AGO1-IP libraries from the fly head.** The format of charts are the same as Figure S7A and B.

**Supplementary Tables**

**Table S1. General profiles of 5’-5N (tab 1) and 5’-8N (tab 2) HISSA libraries (related to Figure 3).** Only reads that were specific to the introduced oligo sequence and of 20nt length were extracted for subsequent analysis. All other reads that were not containing the exact oligo sequence or 20nt were excluded. Poor quality reads had ‘N’s in the sequence readouts and could not be conclusively examined. In the case of 5’-8N HISSA, oligo specific reads must also have the correct fixed region in the 5’ linker in order to be qualified for analysis.

**Table S2. Sequences in the randomized regions of 5’-5N and 5’-8N HISSA (related to Figures 3-5).** The 5’-5N HISSA (tab 1) had 5 randomized positions hence a maximum of 1024 sequences could be obtained. The read count of each sequence is also indicated. 3nt motif enrichment using Bind-n-Seq analysis for 5'-5N HISSA is shown in tab 2. The 64 possible 3nt motifs are arranged in descending order whereby the most enriched motif lies at the top. On the other hand, 5’-8N HISSA (tab 3) had 8 randomized positions that could produce a maximum of ~65000 sequences. Limited by the sequencing depth, most sequences had only 1 read count. Therefore, the sequences were subjected to Bind-n-Seq analysis in search of 3nt motif (tab 4) and also split into 4nt windows (tab 5) for downstream pairwise analysis.

**Table S3. Reads derived from mRNAs used for AGO1-IP enrichment analysis (related to Figure 7).** After applying bioinformatics filters to remove known sRNA species (Figure S11), we considered remaining reads to represent mRNA fragments that are produced from non-hairpin ssRNA molecules. Sheets 2 and 3 contain number of reads and Fisher’s text p-values used for Figure 7.

**Table S4. All oligos used in this study (related to Materials and Methods).** Oligos used for testing the reliability of in vitro loading assay are in tab 1 and those used for demonstrating splinted ligation specificity are in tab 2. All randomized oligos, linkers and RT-PCR primers used for constructing HISSA libraries are in tab 3. Tab 4 contains RNA oligos used for validating HISSA results. Primers used for cloning pEM705-myc-hAGO2 plasmid are listed in tab 5.
